# Lung microbiota-mediated biotransformation of mogroside preserves pulmonary barrier integrity and attenuates PM_2.5_-induced inflammation via NF-κB–Th17 modulations

**DOI:** 10.1038/s41522-026-00992-y

**Published:** 2026-05-05

**Authors:** Kai Wang, Yuan Li, Cuiguang Li, Mohammed Kahiel, Kentaro Nagaoka, Dan Shen, Chunmei Li

**Affiliations:** 1 College of Animal Science and Technology, Nanjing Agriculture University, Nanjing, China; 2 Laboratory of Veterinary Physiology, Department of Veterinary Medicine, Faculty of Agriculture, Tokyo University of Agriculture and Technology, Tokyo, Japan

**Keywords:** Diseases, Microbiology

## Abstract

PM_2.5_-induced lung injury challenges poultry health with limited treatments. Mogroside’s unique therapeutic impact on pulmonary inflammation may involve modulating the lung microbiome, which influences immune function and respiratory health. We first demonstrated that mogroside (MG) supplementation improved growth performance and mitigated PM_2.5_-induced alveolar damage, inflammatory cytokine release, and Th17 differentiation (*p* < 0.05). MG increased the abundance of beneficial bacteria, particularly *Lactobacillus* (*p* < 0.01). Notably, MG IIE accumulated in lung tissues and bronchoalveolar lavage fluid (BALF). To further clarify the role of microbe–metabolite interactions, BALF from MG-treated broilers was transplanted. Only complete BALF containing both MG and microbiota significantly alleviated fibrosis (*p* < 0.05), reshaped lung microbial composition, and modulated metabolites such as taurine and lactic acid. Microbiome analysis identified *Sphingomonas* as a key taxon enriched in MG-BALF, strongly correlated with protective metabolites. In vitro assays confirmed that *Sphingomonas* degraded MG IIE into mogrol via β-glucosidase activity. Finally, a Calu-3–Jurkat T lymphocytes co-culture model revealed that MG IIE, particularly in combination with *Sphingomonas* metabolites, preserved barrier integrity, suppressed NF-κB phosphorylation, reduced ROS, and inhibited Th17-associated cytokine expression. Collectively, MG IIE and its *Sphingomonas*-mediated metabolites form a lung microbiota–metabolite–host axis that protects against PM_2.5_-induced pulmonary injury.

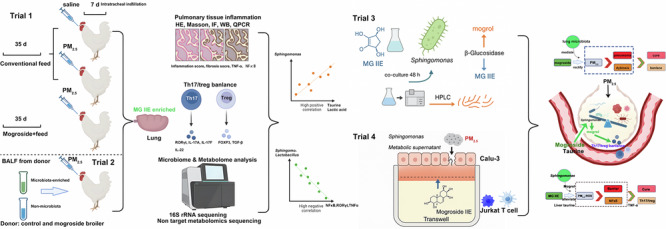

## Introduction

The intensification of poultry farming has led to an increased prevalence of respiratory diseases, which pose significant challenges to poultry health and productivity. Among these, fine particulate matter (PM_2.5_)-induced lung injury has emerged as a critical concern, mainly due to the unique physiological structure of the avian lung, which renders poultry highly susceptible to airborne contaminants^1^. This situation has been exacerbated by recent regulations in China banning antibiotics in animal feed, leading to a sharp rise in respiratory infections that lack effective treatment alternatives^2,3^. PM_2.5_ has been extensively characterized for its physical, chemical, and toxicological properties. It has been implicated in causing severe respiratory inflammation, oxidative stress, and microbial dysbiosis in exposed organisms. Our previous studies characterized PM_2.5_ in detail and its deleterious effects on poultry lung health^4,5^. The lungs host a variety of immune cells, including T helper (Th) cells and regulatory T (Treg) cells, which work in concert to maintain immune homeostasis^6,7^. Disruption of this balance can lead to pulmonary inflammation. Inflammatory lung conditions are often characterized by an increase in the levels of proinflammatory cytokines, such as tumor necrosis factor alpha (TNF-α), interleukin (IL)-6, IL-1β and transforming growth factor-β (TGF-β)^8^. Evidence suggests that proinflammatory cytokines are produced primarily by Th cells, particularly T helper 17 (Th17) cells. In contrast, Treg cells are the main source of anti-inflammatory cytokines. Additionally, an imbalance between Th17 and Treg cells has been strongly linked to the progression of chronic pulmonary inflammation^9^. This imbalance is regulated by critical transcription factors, such as retinoic acid receptor-related orphan receptor-γt (RORγt) and forkhead box P3 (FOXP3), along with cytokines, including IL-6, IL-1β, IL-17A, IL-17F, IL-22, nuclear factor kappa-B (NF-κB), TNF-α, and TGF-β. For example, TNF-α promotes the differentiation and functional enhancement of Th17 cells by increasing the expression of IL-6 and IL-22^10,11^. In addition, TNF-α can also activate transcription factors related to Th17 cells, such as RORγt, which exacerbates lung inflammation. Similarly, NF-κB directly promotes the expression of RORγt and increases the secretion of IL-17. NF-κB inhibits the function of Treg cells by interfering with the expression and stability of Foxp3. In addition, excessive NF-κB signaling may further weaken the immunosuppressive ability of Tregs by enhancing the proinflammatory environment^12^. This dynamic interplay between Th17 and Treg cells is pivotal in controlling lung inflammation and its progression.

Mogroside (MG), derived from *Siraitia grosvenorii*, a traditional Chinese medicinal plant known for its food and therapeutic applications, is a promising intervention^13–15^. MG has been shown to possess potent antioxidative and anti-inflammatory properties in both in vitro and in systemic cellular models^16–19^. Intriguingly, its clinical efficacy appears to be most pronounced in alleviating pulmonary inflammation. Among individual MGs, MG IIE is a diglycosylated, relatively bitter MG that is highly enriched in immature fruit and is regarded as a biogenetic precursor for more highly glycosylated “sweet” MGs (e.g., MG V) during fruit ripening and glycosylation^20,21^. MG IIE is not only a structural intermediate but also a bioactive molecule: recent work reported that mogroside ⅡE alleviated acute lung injury by restraining the Pla2g2a–EGFR axis and downstream AKT/mTOR signaling, supporting its relevance to inflammatory lung disorders^22^. In parallel, accumulating pharmacokinetic and microbiome evidence indicates that mogrosides generally exhibit limited systemic absorption and can undergo stepwise deglycosylation by host enzymes and/or microbiota, yielding less-glycosylated intermediates and ultimately the aglycone mogrol; this implies that local microbial hydrolysis may substantially reshape the exposure profile and bioactivity of MG IIE and related mogrosides. Notably, Different saponins exhibit hydrolytic specificity and functional diversity when utilized by bacteria^23^. This observation raises the question of whether the unique microbiological environment of the lung is pivotal for mediating the therapeutic effects of MG.

Emerging research highlights the importance of lung microbiota in maintaining respiratory health and modulating host responses to environmental insults^24^. The existence of the pulmonary microbiome challenges the long-standing medical perspective that the lungs are sterile. Recent research advancements reveal that changes in the composition and abundance of pulmonary microorganisms may occur even before the clinical symptoms of the disease manifest. Variations in the pulmonary microbiome can influence disease progression and therapeutic outcomes, with certain microbial types being linked to the development of chronic lung diseases^25^. In lung cancer patients, the composition of the lung microbiota significantly differs from that of healthy individuals, and alterations in specific bacterial populations may promote tumorigenesis by modulating immune responses and inflammatory pathways^26^. Furthermore, the pulmonary microbiome influences the progression of chronic respiratory diseases by regulating host immunity and inflammation. In chronic obstructive pulmonary disease, the lung microbiome is associated with various phenotypes and endotypes, and dysbiosis may contribute to the onset of airway inflammation^27^. Unlike the well-studied gut microbiota, the lung microbiota is a relatively nascent field, with many aspects of its composition, function, and interactions with pharmacological agents still poorly understood. Current studies on the relationship between the pulmonary microbiome and lung injury are predominantly based on clinical observations and reviews, with extremely limited experimental evidence. This knowledge gap prompted our investigation into the potential role of the lung microbiota in mediating the therapeutic effects of MG against PM_2.5_-induced lung injury in broilers.

This study was conducted in four stages. First, we evaluated the preventive effects of dietary MG supplementation on PM_2.5_-induced lung injury and lung microbiota dysbiosis. Our findings revealed that MG significantly alleviated pulmonary inflammation symptoms and restored microbial balance, with *Lactobacillus* emerging as a key microbial target. In the second stage, alveolar lavage fluid transplantation was applied to disentangle the respective roles of lung microbiota and MG metabolites. Integrative microbiome–metabolome analysis identified *Sphingomonas* as a key bacterium capable of degrading MGIIE into bioactive compounds. In the third stage, in vitro microbial degradation assays confirmed that *Sphingomonas* metabolized MGIIE into mogrol and related derivatives. Finally, we established a Transwell co-culture model mimicking the pulmonary epithelial barrier to validate the functional relevance of these metabolites. This model demonstrated that PM_2.5_ disrupted epithelial integrity, facilitating MGIIE access to bacterial metabolites, which in turn enhanced its anti-inflammatory and barrier-protective effects.

## Methods

### Animals

**Fig. 1:**
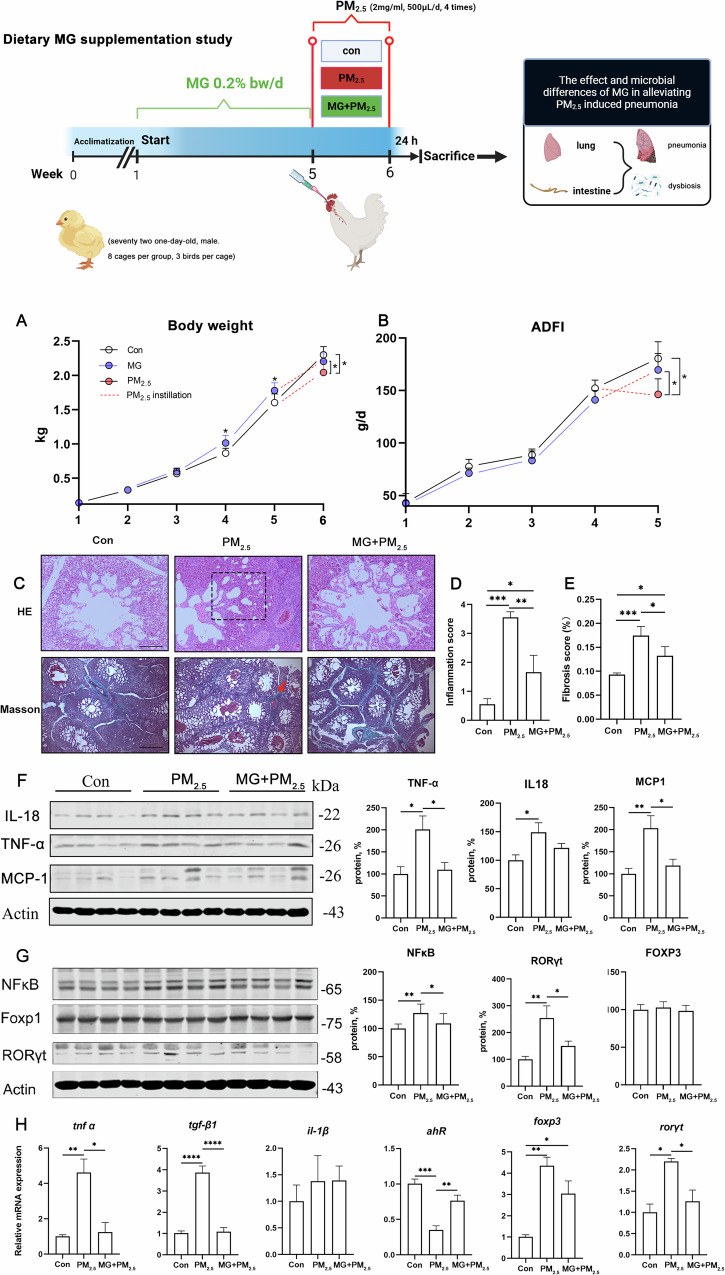
Mogroside on Growth Performance and Lung Tissue Th17 Differentiation in PM_2.5_-induced Broiler Lung Injury. 6-week observation of experimental subjects from different treatment groups. **A** compares the body weight changes among the control group (Con), PM₂.₅ group (P) and mogroside + PM₂.₅ group (MG + PM_2.5_), *n* = 24. **B** presents the comparison of the average daily feed intake (ADFI) among the different treatment groups. **C** Illustrates the histological of lung tissues from the control, P, and MP groups, as shown by H&E and Masson staining, including the degree of damage (**D**) and fibrosis (**E**) in specific regions. **F** Shows the abundance of IL18, TNFα, and MCP1 proteins in lung tissues, (**G**) (**H**): Comparison of Th17/Treg cell-associated transcriptional factors and cytokine’s expression in lung between three different groups. Western blot, protein abundance of NF-κB, FOXP3, RORγt and mRNA level of *Tnf-α, Tgf-β, Il-1β, AhR, Foxp3* and *Rorγt* in lung tissue, *n* = 8. All data are presented as mean ± standard error of the mean (SEM), and statistical analysis was performed accordingly. *p < 0.05, **p < 0.01, ***p < 0.001.

Dietary MG supplementation study: A total of 72 one-day-old male broilers (Arbor Acres, AA) were randomly assigned to three groups: the control (Con) group, the PM_2.5_-induced lung injury (PM_2.5_) group, and the mogroside (High-performance liquid chromatography, HPLC > 98%, CAS: 88901-36-4, Xi’an Zebang Biotechnology Co., Ltd. Chemical structures of mogroside see supplementary materials Figure S1) supplementation (MG) group. Each group contained 24 chickens (8 cages, 3 chickens per cage) housed under identical conditions with ad libitum access to food and water. The broilers in the MG group were fed a basal diet supplemented with mogroside (0.2% w/w)^28^ for 35 days, whereas those in the Con and PM_2.5_ groups received a standard basal diet. On day 35, a PM_2.5_ suspension (500 μl, 2 mg/mL) was instilled into the trachea of chickens in the PM_2.5_ and MG groups to induce localized lung inflammation drip every other day, 4 times. The Con group received sterile saline. The weights, daily weight gains and average daily feed intakes of the chickens were recorded. After 8 days postexposure, all broilers were euthanized under deep anesthesia to minimize stress and ensure humane treatment, in strict accordance with institutional animal welfare guidelines. Specifically, the birds were anesthetized by inhalation of 5% isoflurane using a vaporizer mask. Euthanasia procedures (exsanguination for blood collection and subsequent tissue harvesting) were strictly initiated only after confirming complete unconsciousness and the total loss of protective reflexes. This inhalation method was selected as the rationale to guarantee a rapid, painless induction of anesthesia, thereby eliminating handling stress and preserving the baseline biochemical and immunological parameters in the pulmonary tissues. Lung, liver, serum, intestinal, cecal contents samples and bronchoalveolar lavage fluid (BALF) were collected for further analyses (Fig. 1). The dosage of the PM_2.5_ infusion was calculated on the basis of real-time concentration monitoring in commercial chicken coops. The measured concentration in commercial chicken houses, and the actual concentration in exposure tests. For details, please refer to the supplementary materials Table S1. The current scientific investigation was conducted following the rules and regulations of the ethics committee for the use of animals, Nanjing Agricultural University’s Institute (SYXK(Su)2021-0086), Nanjing, China.

### BALF transplantation

BALF transplantation study: BALF was collected from 35-day-old broilers from two donor groups (*n* = 12): the control group (Normal) and a group fed a mogroside-supplemented diet (MG) for 35 days; BALF samples were subjected to Percoll density-gradient centrifugation to deplete soluble proteins while preserving microbial cells. For details, please refer to the supplementary materials. Each type of BALF was processed into two variants: unfiltered (complete Normal-BALF and complete MG-BALF) and filtered (filtered Normal-BALF and filtered MG-BALF, 0.22μm filter membrane) to distinguish microbial effects. Thirty-six 1-day-old broilers were randomly assigned to six groups (CON, PM_2.5_, complete Normal-BALF, complete MG-BALF, filtered Normal-BALF and filtered MG-BALF), with six birds per group. On day 28, all the groups except the CON group received PM_2.5_ via tracheal instillation (500 μl, 2 mg/mL, tracheal instillation every other day, 4 times). BALF transplantation began on day 35, with recipient groups receiving 500 µL of their respective BALF by tracheal instillation every other day for four doses. The birds were euthanized on day 43 (Fig. 3). After 12 h of tracheal instillation or BALF, all broilers were weighed and humanely euthanized. Lung tissues were photographed and collected for subsequent analyses. Freshly harvested lung tissues were fixed in 4% paraformaldehyde solution for histopathological and immunohistochemical analyses. A portion of the lung tissue was excised for gene and protein expression analyses, rinsed with cold 0.75% normal saline to remove residual blood, snap-frozen in liquid nitrogen, and stored at −80 °C. A portion of the lung tissue was used for microbiota sequencing and nontargeted metabolomics to evaluate the microbial and metabolic composition.

### Hematoxylin and eosin (HE) staining

Fresh 1 cm^3^ lung tissues were collected and fixed in 4% paraformaldehyde for 24 hours to preserve their structure. Then subjected to ethanol gradients for dehydration, cleared using xylene, and embedded in paraffin. Thin sections measuring 5 µm in thickness were sliced with a microtome and affixed to glass slides. The sections underwent deparaffinization and rehydration before being stained with hematoxylin for 5 min and eosin for 2 min. Afterward, the slides were dehydrated again, cleared, and sealed with a resin-based mounting medium under coverslips. Lung histopathological scoring was performed to evaluate tissue damage and inflammatory changes. Five nonoverlapping fields per section were randomly selected and examined under a light microscope at 200× magnification (OLYMPUS BX51, Japan). The scoring criteria included alveolar structure integrity, degree of pulmonary edema, inflammatory cell infiltration, and extent of fibrosis. Each parameter was assigned a score ranging from 0 to 4, where 0 indicated no abnormality and 4 represented severe pathological changes. Scores were averaged across fields for each sample. Two independent blinded observers conducted all scoring to minimize bias, and discussions resolved discrepancies.

### Masson staining

Lung tissue sections were deparaffinized, rehydrated, and subjected to Masson’s trichrome staining to evaluate fibrosis. In the first experiment, the collagen fibers were stained green, whereas in the second experiment, they were stained blue. The sections were counterstained with hematoxylin for visualization of the nuclei. The stained sections were observed under a light microscope, and representative images were captured (OLYMPUS BX51, Japan). ImageJ software (version: 1.54 f, USA) was used to quantify the fibrotic area. The digital images of the stained sections were converted to 8-bit grayscale, and the threshold tool was applied to isolate the fibrotic regions. The percentage of fibrosis was calculated as the ratio of the fibrotic area to the total tissue area. Measurements were performed on five randomly selected fields per section at 200× magnification, and the results were averaged for each sample.

### Immunohistochemical staining

Immunohistochemical staining was carried out on 4-µm-thick paraffin-embedded lung tissue sections. The sections underwent deparaffinization using xylene and were then rehydrated through a graded ethanol series. Antigen retrieval for 20 min. To inhibit endogenous peroxidase activity, the sections were treated with 3% hydrogen peroxide for 10 minutes at room temperature. Following this, nonspecific binding was blocked with 5% bovine serum albumin for 30 min. The sections were incubated overnight at 4 °C with primary antibodies against FOXP1 (Mouse-derived FOXP1 antibody, 92% homologous to chicken FOXP3, dilution 1:100, A12685, ABclonal, China) and RORγt (dilution 1:200, A10240, ABclonal, China). Subsequently, biotinylated secondary antibodies were applied, and the avidin-biotin-peroxidase complex method was used for visualization. The immunoreactivity was visualized by applying 3,3’-diaminobenzidine substrate, followed by counterstaining with hematoxylin. Integrated optical density values were measured via ImageJ software (version: 1.54 f, USA) for quantification. Randomly selected fields (8 pulmonary lobules per slide) from 4 tissue sections per group were analyzed to determine protein expression levels.

### Western blot

To analyze the expression of IL-18, TNF-α, FOXP3, RORγt, IL-6, MCP1, and NF-κB in lung and intestinal tissues, Western blotting was employed. Briefly, tissue samples were homogenized and bicinchoninic acid (BCA) quantified. Equal amounts of protein (50 µg per sample) were resolved by 10–12% sodium dodecyl sulfate-polyacrylamide gel electrophoresis and subsequently transferred onto polyvinylidene fluoride membranes. Blocking was performed using 5% nonfat milk in Tris-buffered saline with Tween-20 1 h. Primary antibodies specific for IL-18 (1:500, 10663-1-AP, Proteintech, USA), TNF-α (1:500, HZ-1014, Proteintech, USA), FOXP1 (1:1000, A12685, ABclonal, China), RORγt (1:1000, A10240, ABclonal, China), IL-6 (1:1000, 26404-1-AP, Proteintech, USA), MCP1 (1:500, 26161-1-AP, Proteintech, USA), and NFκB (1:1000, 10745-1-AP, Proteintech, USA) incubated with membranes at 4 °C for 12 h. After washing, the membranes were incubated with horseradish peroxidase-conjugated secondary antibodies (1:5000, HRP-60004, Proteintech) for 1 h at room temperature. Protein bands were visualized using an enhanced chemiluminescence (ECL) detection system (Bio-Rad ChemiDoc XRS + , USA), and their intensities were analyzed using ImageJ software (version 1.54 f, USA). GAPDH and β-actin (10494-1-AP, Proteintech, USA) served as internal controls to ensure equal protein loading.

### Quantitative real-time polymerase chain reaction (qPCR)

Quantitative PCR (qPCR) was utilized to evaluate the mRNA expression levels of target genes in lung. Total RNA was isolated from lung tissues using TRIzol reagent (Takara, Japan). The RNA concentration and purity were determined using a spectrophotometer. cDNA was synthesized from 1 µg of total RNA using a reverse transcription kit (Bio-Rad, Hercules, CA, USA). SYBR Green PCR Master Mix and gene-specific primers targeting *Muc1, Tnf-α, Tgf-β, Il-1β, Ahr, Il-17a, Il-17f, Il-22, Foxp3*, and *Rorγt* (primer sequences are provided in Table S2). Amplification was conducted on a QuantStudio 7 Flex Real-Time PCR System (ABI, USA). Relative gene expression levels were calculated using the 2^-^^ΔΔCt^ method, with *β-actin*.

### 16S rRNA gene sequencing and analysis

Lung tissue and cecal contents samples from six randomly chosen broilers in each group were subjected to 16S rRNA gene sequencing. Total genomic DNA was extracted from lung tissues via the sodium dodecyl sulfate (SDS) method. The hypervariable V4 region of the bacterial 16S rRNA gene was amplified with the barcoded primers 515 F (5′-GTGCCAGCMGCCGCGGTAA-3′) and 806 R (5′-GGACTACHVGGGTWTCTAAT-3′). Sequencing libraries were prepared via the Ion Plus Fragment Library Kit (Thermo Scientific, USA) and sequenced on the Ion S5™ XL platform (Thermo Fisher, USA), generating 400–600 bp single-end reads. Operational taxonomic units (OTUs) were determined by clustering sequences with 97% similarity. The α diversity of the microbial communities, including the observed species, ACE, Chao1, Shannon, and Simpson indices, was calculated via QIIME (version 1.9.1) to evaluate the species diversity within each sample. Differences in microbial composition between groups (β diversity) were explored via methods such as principal coordinate analysis (PCoA) and hierarchical clustering. These analyses provided a visual representation of species diversity and group-specific differences. Additional methodological details are available in the Supplementary Materials. Sequences were clustered into 97% similarity OTUs for downstream community- and genus-level comparisons; given the strong effect sizes observed in dominant taxa and the multi-layer validation in this work, we did not reprocess reads into ASVs in the present revision.

### Nontargeted metabolomics

To explore the metabolic alterations associated with PM_2.5_-induced changes in the host lung microenvironment and microbiome, pulmonary metabolomics was performed. Six lung tissue samples were randomly selected from each group for metabolite profiling. Lung tissues (100 mg) were pulverized in liquid nitrogen, and the resulting homogenates were resuspended, centrifuged, and subjected to further dilution and filtration. After an additional centrifugation step, the processed supernatant was prepared for liquid chromatography‒mass spectrometry (LC‒MS) analysis. The UHPLC‒MS/MS analysis data were processed via Compound Discoverer 3.0 (CD3.0, Thermo Fisher, USA). Metabolites were identified and annotated through the Kyoto Encyclopedia of Genes and Genomes (KEGG) database. Partial least squares discriminant analysis (PLS-DA) was used to differentiate between sample groups on the basis of principal components. Differentially abundant metabolites were identified on the basis of the following criteria: Data visualization included heatmaps to highlight variations in metabolite levels, whereas Pearson correlation analyses were conducted to evaluate relationships among differentially abundant metabolites, with statistical significance set at *p* < 0.05. Metabolite functions and pathways were further analyzed via the Kyoto Encyclopedia of Genes and Genomes (KEGG) database to uncover their biological roles. Receiver operating characteristic (ROC) curves were generated to identify potential metabolite biomarkers via R software (version 3.4.3). The supplementary materials provide additional details on the methodology.

### Bacterial culture and sample collection

In vitro microbial degradation assay: *Sphingomonas sp*. (obtained in lyophilized form from CICC 24503) was revived in sterile R2A broth and incubated at 30 °C for 24 h. After activation, cells were harvested by centrifugation (5000 × *g*, 10 min), washed twice with sterile phosphate-buffered saline (PBS, pH 7.2), and resuspended to a final density of 1 × 10^7^ CFU/mL in PBS-based culture medium. The bacterial suspension was divided into two groups: (1) *Sphingomonas* alone (SP), and (2) *Sphingomonas* supplemented with mogroside IIE (SP + MIIE) at a final concentration of 500 µg/mL. Mogroside IIE (purity ≥98%, 88901-38-6, TZ biological company, Wuhan China) was dissolved in dimethyl sulfoxide (DMSO) and sterilized by 0.22 µm filtration before addition; the final DMSO concentration in all cultures was adjusted to 0.1% (v/v). Cultures (10 mL per tube) were incubated at 37 °C without shaking for up to 48 h. Samples were collected at 0, 6, 12, 24, and 48 hours, with six independent replicates per group per time point (*n* = 6). At each time point, 1 mL of culture was withdrawn: 200 µL was used immediately to measure optical density at 600 nm (OD₆₀₀) using a spectrophotometer (BioTek Epoch 2), and 800 µL was centrifuged at 12,000 × *g* for 10 min at 4 °C. Supernatants were aliquoted and stored at −80 °C for subsequent biochemical analyses. pH was measured directly from fresh supernatant using a calibrated digital pH meter (Mettler Toledo FE28) with temperature compensation. For HPLC analysis, mogroside IIE and its aglycone mogrol were extracted from 500 µL of culture supernatant via solid-phase extraction using preconditioned Oasis HLB cartridges (Waters, USA), eluted with methanol, evaporated under nitrogen, and reconstituted in 100 µL of 30% methanol. Samples were analyzed on an Agilent 1260 HPLC system equipped with a C18 column (4.6 × 250 mm, 5 µm), using a mobile phase of 0.1% formic acid in water (A) and acetonitrile (B) under gradient conditions (25–80% B over 20 min), with detection at 254 nm. Quantification was performed using standard curves constructed from serial dilutions of mogroside IIE and mogrol (0.1–10 µg/mL). β-Glucosidase activity in the culture supernatants was measured using p-nitrophenyl-β-D-glucopyranoside (pNPG, Sigma-Aldrich) as the substrate. In brief, 50 µL of culture supernatant was mixed with 50 µL of 5 mM pNPG (in 50 mM citrate-phosphate buffer, pH 6.0) and incubated at 37 °C for 30 min. The reaction was terminated by adding 100 µL of 1 M Na₂CO₃, and absorbance at 405 nm was measured using a microplate reader. Enzyme activity was calculated based on a standard curve of p-nitrophenol (pNP) and expressed as µmol pNP released per minute per mg protein (µmol/min/mg). Protein concentrations were determined using a BCA protein assay kit (Thermo Fisher Scientific) according to the manufacturer’s protocol.

### Cell culture and experimental design

In vitro co-culture mechanistic validation: Calu-3 bronchial epithelial cells were seeded onto Transwell inserts and cultured for 8 days to allow differentiation and formation of a polarized epithelial barrier, as confirmed by transepithelial electrical resistance (TEER) measurements. Jurkat T lymphocytes were seeded in the lower chamber to mimic immune cell responses. In the first phase, Calu-3 cells were exposed to PM_2.5_ (25, 50, 100 μg/mL), bacterial metabolites (BM, 1%, 2%, 5%) or MG IIE (25, 50, 100 μg/mL) to evaluate cytotoxicity and optimal concentrations via Cell Counting Kit-8 (CCK-8) assay. In the second phase, mature Calu-3 monolayers were challenged with PM_2.5_ or PM_2.5_ + BM (live or heat-inactivated) in the apical chamber, while MG IIE was added to the basolateral chamber. Barrier integrity was assessed by TEER. In the third phase, mechanistic studies were conducted with groups of Con, PM_2.5_, PM_2.5_ + NF-κB inhibitor (BMS-345541), and PM_2.5_ + NF-κB inhibitor +MG IIE + BM, with assessment of total NF-κB, phosphorylated IκB kinase β (IKKβ) and NF-κB, nuclear translocation, and NF-κB–dependent cytokines. More information see in supplement materials. All in vitro experiments were performed in six independent biological replicates (*n* = 6) per group, and the entire study was repeated three times to ensure reproducibility.

### Statistical analysis

Statistical analyses were conducted via GraphPad Prism version 9.00 (GraphPad Software, LLC, CA). Initially, one-way analysis of variance (ANOVA) was performed to assess significant differences among the six groups in BALF transplantation study, with Group PM_2.5_ serving as the baseline for comparisons. Two-way ANOVA was subsequently applied to evaluate interactions and differences, between Groups normal and MG. All the data are presented as the means ± standard errors of the means (SEMs). Pearson correlation coefficients were calculated to quantify the degree of linear relationships between variables. A *p-*value < 0.05 was considered indicative of statistically significant differences.

## Results

### Mogroside on the Growth Performance of Broilers Exposed to PM_2.5_

At week 4, the body weight of the broilers fed mogroside (MG) was greater than the control group (*p* < 0.05), and this difference persisted until week 5, when PM_2.5_ was instilled (Fig. 1A). At 6 weeks of age, no significant difference in body weight was observed between the MG and control groups (Fig. 1A). In contrast, the body weight of the PM_2.5_-treated group was lower than that of both the control and MG groups at week 6 (Fig. 1A, *p* < 0.05). Week after PM_2.5_ instillation, the average daily feed intake (ADFI) of the PM_2.5_ group was reduced (Fig. 1B, *p* < 0.05).

### Mogroside significantly alleviated PM_2.5_-induced lung damage and inflammation but had no significant effect on the intestinal

Histopathological examination of lung tissues revealed that the PM_2.5_ group exhibited significant inflammatory responses, with severe alveolar structural damage, extensive infiltration of inflammatory cells, and widespread fibrotic staining (Fig. 1C). In contrast, the lung tissue of the MG group appeared relatively intact with milder inflammatory reactions, indicating that MG alleviated PM_2.5_-induced lung tissue damage and fibrosis (Fig. 1D, E, *p* < 0.05). The protein levels of the pro-inflammatory cytokines TNF-α, IL-18, and MCP-1 were markedly upregulated, whereas MG significantly alleviated the upregulation of TNF-α and MCP-1(Fig. 1F, *p* < 0.05). PM_2.5_ did not induce an increase in intestinal inflammatory cytokine levels (Fig. S2).

### Mogroside Attenuates PM_2.5_-Induced Th17 Differentiation and Inflammatory Responses

*Tgf-β, Tnf-α, Foxp3*, and *Rorγt*, as well as proteins such as NF-κB and RORγt, was upregulated in the PM_2.5_ group compared with both the control and MG groups (Fig. 1G, H, *p* < 0.05). In contrast, the MG group presented expression levels of most genes and proteins that were comparable to those of the control group but downregulated relative to those of the PM_2.5_ group (*p* < 0.05). No significant differences were observed in the expression levels of *Il-1β* and FOXP3 across the groups (Fig. 1G, H).

### Mogroside modulates lung microbial composition and reduces the abundance of proinflammatory pathogens

**Fig. 2:**
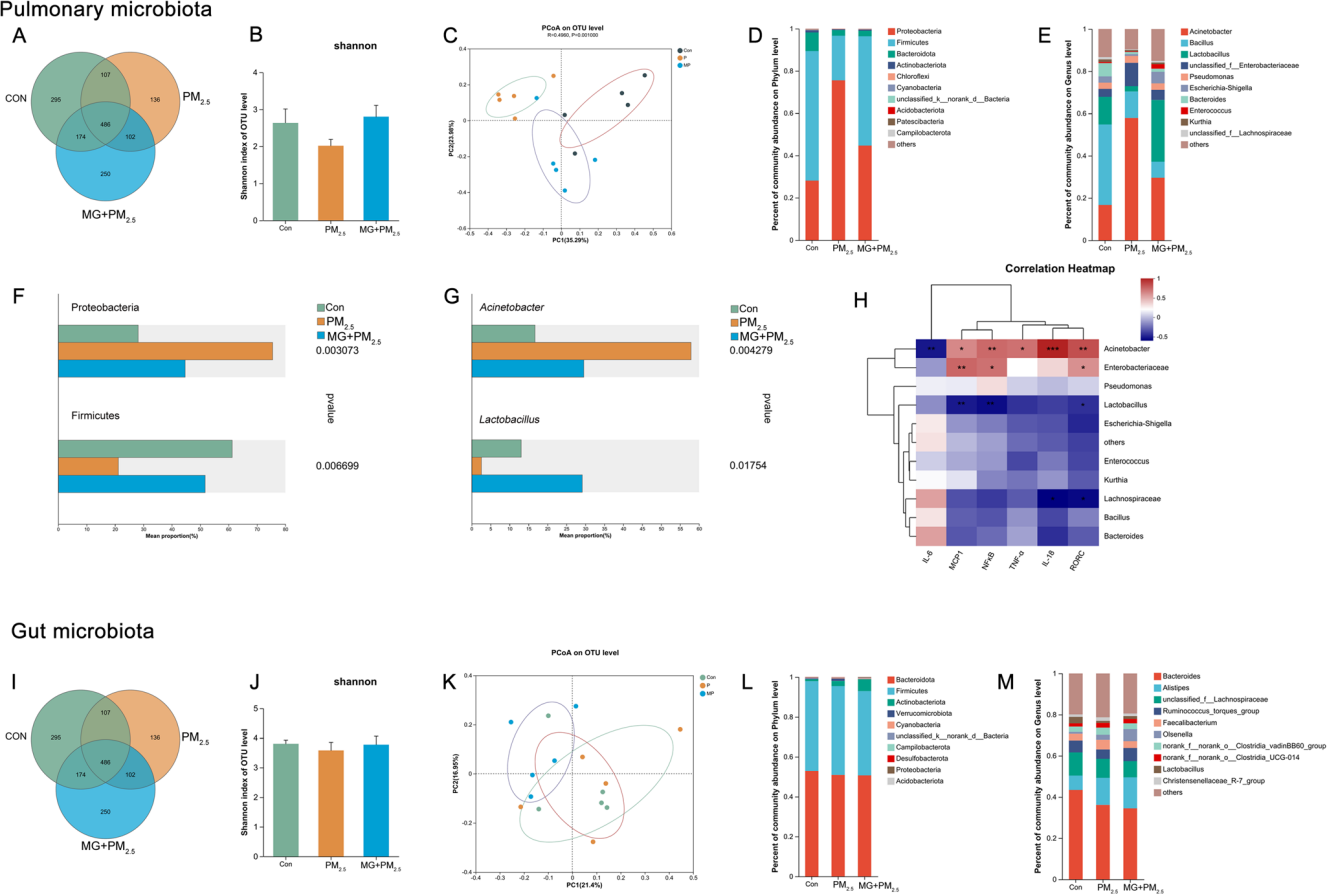
Mogroside on Lung and gut microbial communities in PM_2.5_-induced Broiler Lung Injury. **A** The comparison of lung microbiota (Venn) between the three groups. **B**, **C** The comparison of lung microbiota α-diversity and β-diversity between the three groups. **D**, **E** At the phylum and genus level, the comparison of relative abundance between the three groups. **F**, **G** Differential bacteria at the phyum and genus level. **H** Correlation between bacteria and Th17 inflammatory factors. **I** The comparison of gut microbiota (Venn) between the three groups. **J**, **K** The comparison of gut microbiota α-diversity and β-diversity between the three groups. **L**, **M** At the phylum and genus level, the comparison of relative abundance between the three groups. *p < 0.05, **p < 0.01, ***p < 0.001, n = 5.

The Venn diagram (Fig. 2A) shows that PM_2.5_ exposure reduced the total number of OTUs compared to the Control group, indicating a loss of microbial richness. However, MG supplementation effectively restored the OTU count (Fig. 2A). No significant differences in α-diversity were detected among the three groups (Fig. 2B); however, β-diversity significantly varied (Fig. 2C). At the phylum level, differences were primarily noted in the relative abundances of Firmicutes and Bacteroidetes (Fig. 2D), whereas at the genus level, changes were evident in the abundances of *Acinetobacter*, a major respiratory pathogen, and *Lactobacillus*, a beneficial bacterium (Fig. 2E). PM_2.5_ exposure significantly increased the relative abundance of Proteobacteria (*p* < 0.01) while markedly reducing the abundance of Firmicutes (*p* < 0.01) compared to the Control group. However, MG supplementation effectively reversed this dysbiosis, significantly downregulating Proteobacteria and restoring Firmicutes levels to values comparable to the Control group (Fig. 2F). Compared with the PM_2.5_ treatment, MG increased the abundance of *Lactobacillus* (*p* < 0.05) and decreased the abundance of *Acinetobacter* (Fig. 2G, *p* < 0.05). linear discriminant analysis effect size (LEfSe) revealed that *Lactobacillus* made the greatest contribution across taxonomic levels, from phylum to genus, within the MG-treated lung microbiota (Fig. S3A). Notably, *Acinetobacter* was significantly positively correlated with the proinflammatory factors MCP1, NF-κB, TNF-α, IL-18, and RORγt, whereas *Lactobacillus* was significantly negatively correlated with MCP1, NF-κB, and RORγt (Fig. 2H). Furthermore, MG was unable to reverse the PM_2.5_-induced changes in the diversity and richness of the gut microbiota (Fig. 2I-M).

### BALF transplantation in PM_2.5_-induced pulmonary fibrosis and inflammation

**Fig. 3:**
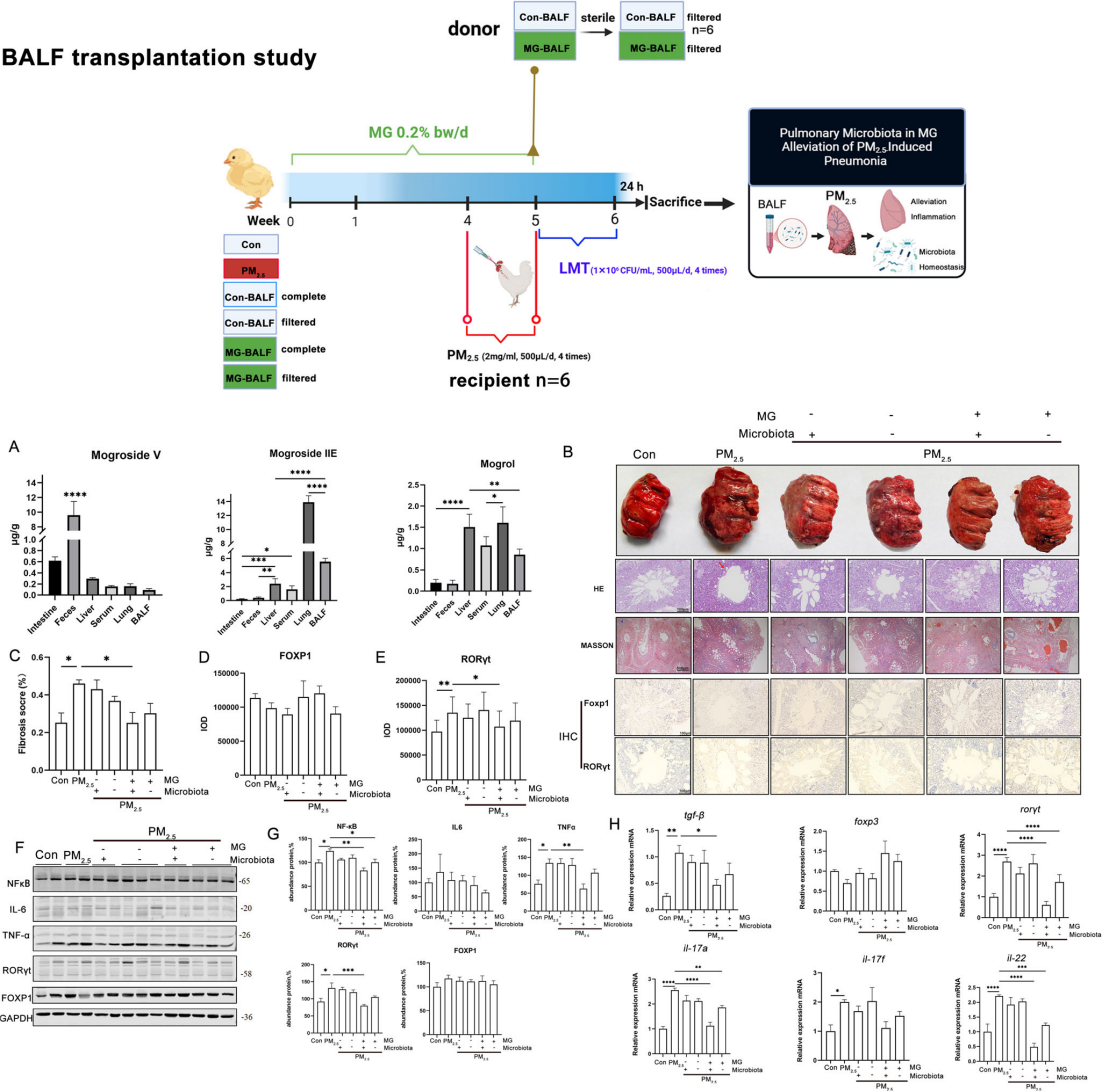
Bronchoalveolar lavage fluid transplantation on Lung Tissue and Th17 Differentiation in PM_2.5_-induced Broiler Lung Injury. 8 days observation of experimental subjects from different treatment groups. BALF was collected from 35-day-old broilers from two donor groups: the control group and a group fed a mogroside-supplemented diet for 35 days. Each type of BALF was processed into two variants: unfiltered (BALF with microbiota) and filtered (BALF without microbiota) to distinguish microbial effects, *n* = 6. **A** In vivo distribution of mogrosides of dietary MG supplementation study, *n* = 8. **B** Illustrates the histological and th17 differentiation protein of lung tissue among the six group by HE, masson and immunohistochemistry (IHC). Scale bars, 100 μm. IOD, integrated optical density. **C** Fibrosis scores (**D**, **E**): Semi-quantitative IHC scoring of FOXP1 and RORγt protein expression in lung. **F**, **G** Abundance of NF-κB, IL6, TNFα, RORγt and FOXP3 proteins in lung tissues. **H** The comparison of mRNA expression level of *Tgf-β, Foxp3, Rorγt, Il-17a, Il-17f* and *Il-22* in the lung tissue among groups. *n* = 6. All data are presented as mean ± standard error of the mean (SEM), and statistical analysis was performed accordingly (One-way ANOVA only vs. PM_2.5_ group), *p < 0.05, **p < 0.01, ***p < 0.001.

The absorption rate of mogroside V was very low, with the majority excreted via feces. Mogroside IIE (MG IIE) predominantly accumulated in the lung tissue and bronchoalveolar lavage fluid, whereas mogrol was abundantly distributed in the liver, serum, lung, and bronchoalveolar lavage fluid (Fig. 3A). Histopathological examination of lung tissue revealed that, compared with control, PM_2.5_ exposure induced pronounced pulmonary edema and alveolar expansion, accompanied by diffuse disruption of lobular structures and significantly greater fibrosis levels (Fig. 3B). Significant attenuation of PM_2.5_-induced lung injury was observed only with the complete MG-BALF (Fig. 3C).

### BALF Transplantation on PM_2.5_-induced Pulmonary Th17 Differentiation

Immunohistochemical analysis of lung tissue revealed that the abundance of RORγt in the PM_2.5_ group greater than that in the control and MG-BALF groups (*p* < 0.05), whereas the abundance of FOXP3 protein was not significantly different (Fig. 3B, D, E). Compared with the control, MG-BALF groups, the PM_2.5_ group presented upregulation of proinflammatory proteins, including NF-κB, TNFα, and RORγt (Fig. 3F, G, *p* < 0.05). However, no significant differences were observed in the abundance of the IL-6 and FOXP3 proteins (Fig. 3G). The transcriptional analysis revealed significant regulatory effects of MG and the microbiota on key inflammatory and immune markers. PM_2.5_ exposure markedly increased the mRNA expression of *tgf-β, il-17a, il-22* and *rorγt* compared with the control group (Fig. 3H, *p* < 0.0001). However, *Foxp3* expression did not significantly differ among the groups.

**Fig. 4:**
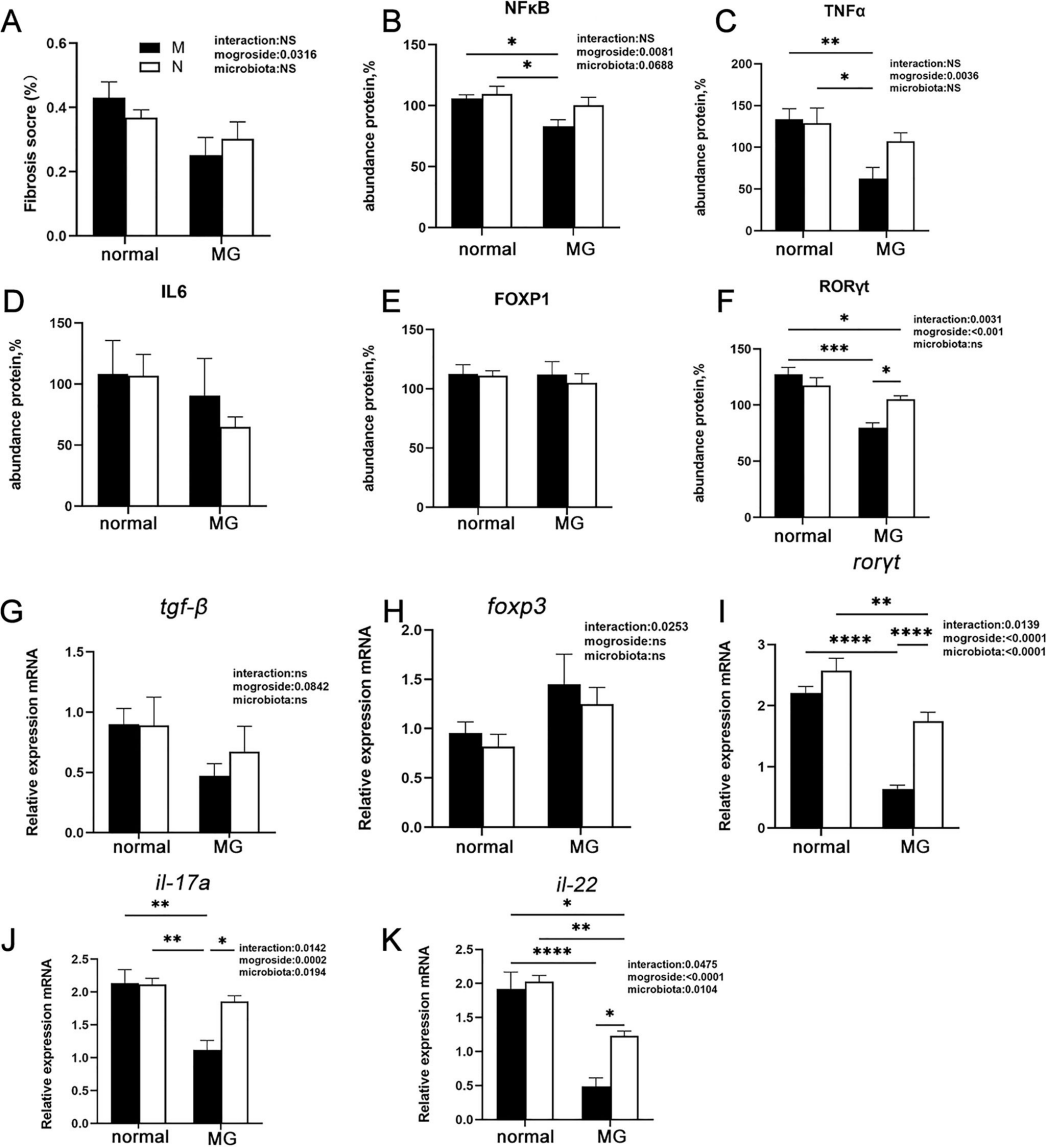
Bronchoalveolar lavage fluid transplantation on PM_2.5_-Induced Th17 Differentiation and Inflammatory Responses. **A**–**F** Fibrosis scores, the comparison of protein expression level of NF-κb, TNF-α, IL-6, FOXP1, RORγt; (**G**–**K**)*Tgf-β, Foxp3, Rorγt, Il-17a, Il-17f* and *Il-22* in the lung tissue among groups, two-way ANOVA analysis, normal (control donor BALF without mogroside), MG (donor BALF with mogroside), M: unfilter (BALF with microbiota), N: filter (BALF without microbiota), Control: *n* = 5, other groups: *n* = 6. *p < 0.05, **p < 0.01, ***p < 0.001, ****p < 0.0001.

Fibrosis scoring further demonstrated a significant main effect of MG supplementation when comparing the normal-BALF group to the MG-BALF group (Fig. 4A). Two-way factor analysis revealed that NF-κB and TNF-α levels were lower in the MG-M group than in the normal-M and normal -N groups (Fig. 4B, C, *p* < *0.05*). Furthermore, the RORγt abundance in the MG-M group was lower than that in both the normal and MG-N groups (Fig. 4F, *p* < 0.05). BALF containing either MG and microbiota or only MG-microbiota significantly attenuated this PM_2.5_-induced *rorγt*, *il-17a* and *il-22* upregulation, with complete MG-BALF showing a stronger suppressive effect (Fig. 4I–K, *p* < 0.05).

### BALF Transplantation on Lung Microbiota Composition in PM_2.5_-Induced Dysbiosis

**Fig. 5:**
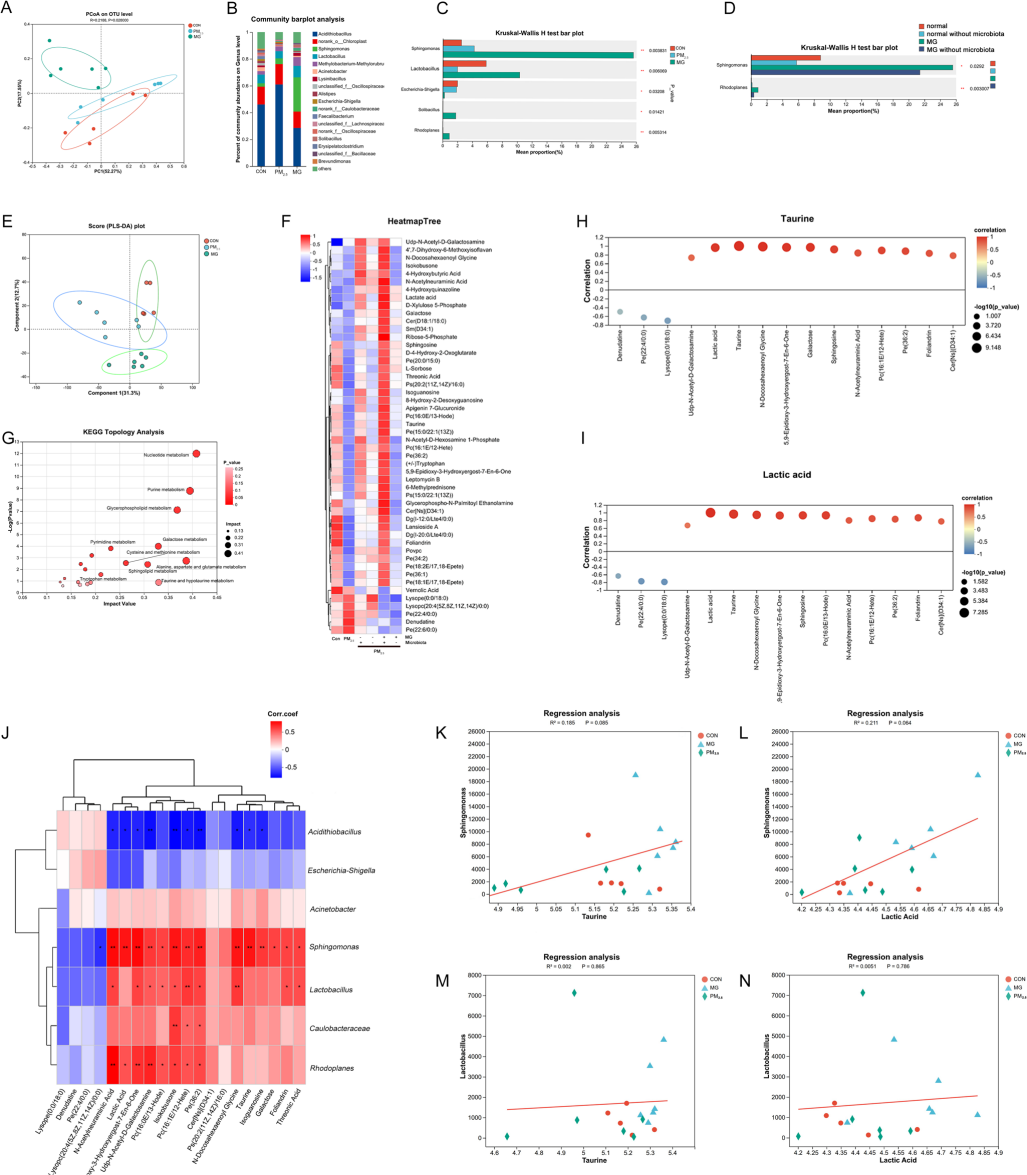
Bronchoalveolar lavage fluid transplantation on Lung microbial communities and metabolite profile in PM_2.5_-induced Broiler Lung Injury. **A** The comparison of lung microbiota β-diversity. **B** At the genus level, the comparison of relative abundance between the the con, P and MB groups. **C** Differential bacteria at the genus level. **D** Differential bacteria at the genus level between the normal BALF, normal -BALF without microbiota, MG-BALF and MG-BALF without microbiota groups. **E**: Analysis of metabolite differences between PLS-DA. **F** The comparison of heat map of different metabolites between the all groups. **G** KEGG pathway enrichment analysis, each bubble represents a KEGG pathway, with size indicating impact value, and the x-axis showing relative importance, while the y-axis reflects enrichment significance (-log10(P-value)). **H**, **I** Metabolite correlation analysis, Taurine and Lactic acid. Red dots for positive and blue for negative correlations, darker colors indicating stronger correlations, and larger dots representing smaller *P*-values. **J** Correlation between differential metabolites and differential bacteria. **K**, **L** Correlation of *Sphingomonas* with taurine and lactic acid, respectively. **M**, **N** Correlation of *Lactobacillus* with taurine and lactic acid, respectively. Red dots for positive and blue for negative correlations, darker colors indicating stronger correlations, and larger dots representing smaller P-values. *p < 0.05, **p < 0.01, C: *n* = 5, other groups: *n* = 6.

After filtration treatment, the bacterial abundance in the BALF decreased by approximately 10^3^ (Fig. S4A, *p* < 0.001). The microbiota analysis revealed significant shifts in the groups’ lung bacterial community diversity and composition. The Chao index (Fig. S4B) indicated that genus-level richness was lower in the PM_2.5_ group than in the control group, with partial recovery observed in the complete MG-BALF group. PCoA based on the OTU level (Fig. 5A) revealed distinct clustering between the CON, PM_2.5_, and complete MG-BALF groups, with differences in microbial structure among the groups (*p* = 0.0200). Community bar plot analysis (Fig. 5B, C) revealed altered genus-level abundance profiles, with an increase in pathogenic taxa such as *Acidithiobacillus* and *Escherichia-Shigella* in the PM_2.5_ group, whereas beneficial taxa such as *Lactobacillus* were more abundant in the complete MG-BALF group. Pairwise comparisons (Fig. 5D) further highlighted differences in *Sphingomonas* abundance between the normal-BALF and MG-BALF groups (*p* = 0.0292 and *p* = 0.003007, respectively). Relative to PM_2.5_ group, the abundance of *Acidithiobacillus* gradually decreased with BALF transplantation in normal-BALF and MG-BALF, but MG only BALF was higher than that in complete MG-BALF group (Fig. S4C). LEfSe analysis (Fig.S4E) revealed distinct biomarkers enriched in the CON, PM_2.5_, and MG-BALF groups, with *Sphingoomonas* emerging as key discriminatory taxa in MG-BALF group.

### BALF transplantation on metabolic alterations associated with PM_2.5_-induced dysbiosis

The metabolic profiles of the lung microbiota in the different treatment groups were analyzed via PLS-DA, KEGG topology analysis, heatmap clustering and metabolite correlation analysis. The PLS-DA plot revealed distinct clustering patterns among the groups, indicating significant differences in metabolite compositions (Fig. 5E). Heatmap analysis revealed distinct metabolite abundances, with several metabolites, including lactic acid, galactose, taurine, Udp-N-acetyl-D-galactosamine and sphingosine, showing prominent differences between the complete MG-BALF group and the other groups (Fig. 5F). In addition, complete MG-BALF was significantly separated from the metabolites in the MG-BALF group (Fig. S5A). KEGG pathway enrichment revealed that taurine metabolism, glycerophospholipid metabolism, purine metabolism, and galactose metabolism were the most impacted pathways (Fig. 5G), particularly in the complete MG-BALF group, compared with the PM_2.5_ group (Fig. S5 B). Taurine metabolism was only enriched in the MG containing group (Fig. S5C–H). These metabolites also had a significant positive correlation (Fig. 5H, I). Analysis identified key metabolites such as taurine and lactic acid as major contributors to the separation between PM_2.5_ and MG group, further emphasizing the metabolic reprogramming induced by BALF transplantation from MG-treated broilers.

### Correlation analysis between metabolites and bacteria

Heatmap showing the correlations between bacterial genera and metabolites, where different colors indicate the direction and strength of the correlation. The results revealed that *Sphingomona*s was significantly positively correlated with metabolites such as lactic acid, taurine, and galactose, whereas *Acidithiobacillus* was significantly negatively correlated with these metabolites. *Lactobacillus* had weaker correlations with these metabolites but stronger associations with other metabolites (Fig. 5J). These relationships were further explored through regression analysis: a strong positive correlation was observed between *Sphingomonas* and taurine as well as lactic acid (Fig. 5K, L), whereas the correlation between *Lactobacillus* and these metabolites was not significant (Fig. 5M, N).

### Mogroside IIE enhances hepatic taurine and is degraded by *Sphingomonas*

**Fig. 6:**
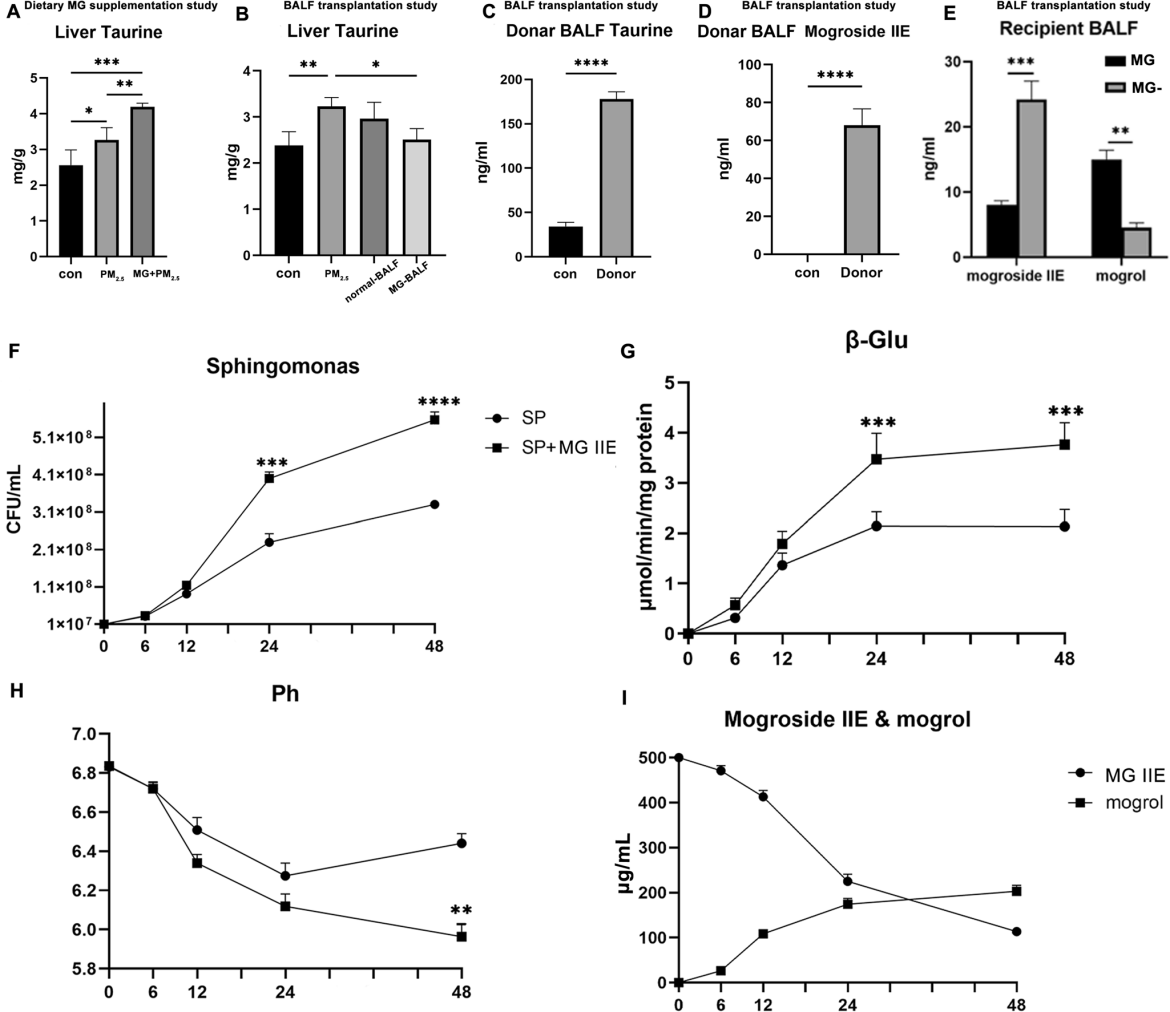
Taurine elevation and microbial degradation of mogroside IIE in vivo and in vitro. **A**, **B** Taurine concentration in the liver of MG-treated broilers in dietary MG supplementation study and BALF transplantation study. **C**, **D** Concentrations of taurine and MG IIE in donor BALF from BALF transplantation study. **E** Concentrations of MG IIE and mogrol in recipient BALF at the end of BALF transplantation study. In vitro degradation kinetics of mogroside IIE by *Sphingomonas* over 48 h. **F** Bacterial growth curve (OD₆₀₀), (**G**) β-glucosidase activity in the culture supernatant, and (**H**) pH changes. (**I**) HPLC-based quantification of mogroside IIE and its hydrolysis product mogrol during co-culture with *Sphingomonas*. Data are presented as mean ± SD (*n* = 6). *p* < 0.05 indicates statistical significance.

In dietary MG supplementation study, taurine levels in the liver were significantly elevated following MG administration (Fig. 6A, *p* < 0.05). In BALF transplantation study, BALF transplantation did not alter hepatic taurine levels compared with the control group (Fig. 6B, *p* < 0.05). Notably, taurine and MG IIE abundances were significantly higher in the donors of BALF transplantation study than in the controls (Fig. 6C, D*p* < 0.05). Furthermore, compared with filtered MG-BALF recipient, complete MG-BALF exhibited markedly decreased MG IIE levels but significantly increased mogrol levels (Fig. 6E, *p* < 0.05). Figure 6A–D depict the in vitro degradation kinetics of MG IIE by *Sphingomonas* over a 48-hour period. Compared to the untreated control group, MG IIE treatment significantly promoted bacterial growth (Fig. 6F), accompanied by an increase in β-glucosidase activity in the culture supernatant (Fig. 6G) and a gradual decrease in pH (Fig. 6H). In Fig. 6I, HPLC quantification further confirmed a time-dependent reduction in MG IIE, along with a corresponding increase in mogrol during co-culture with *Sphingomonas*, supporting the enzymatic hydrolysis of MG IIE.

### MG IIE and Microbial Metabolites Preserve Epithelial Integrity and Modulate NF-κB–Th17 Signaling

**Fig. 7:**
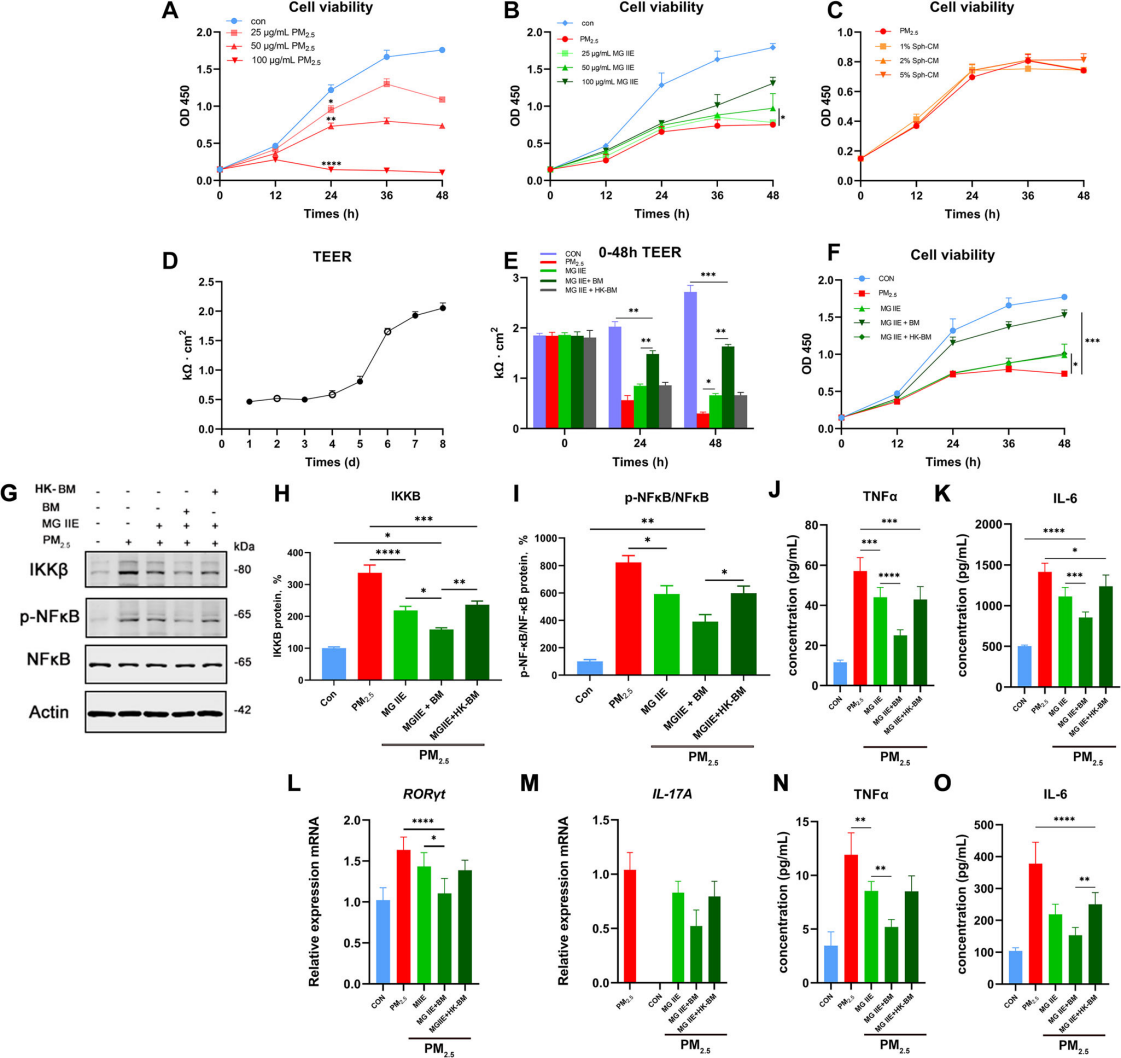
Effects of PM_2.5_, MG IIE, *Sphingomonas* metabolic supernatant (BM) and their respective treatments on a Calu-3 and Jurkat T cell co-culture system. Time-dependent impact of various concentrations of PM_2.5_ (**A**), MG IIE (**B**), BM (**C**) on Calu-3 cell viability, measured CCK8 via OD450. **D** The trans-epithelial electrical resistance (TEER, Ω·cm²) of the Calu-3 cell monolayer measured daily over 8 days, with white circles indicating medium changes. **E** TEER values at 0, 24, and 48 h for Calu-3 cells under different treatment conditions: CON (Control), PM_2.5_, MG IIE, MG IIE + BM and heat-kill (HK-BM), under the combined treatment of PM_2.5_. **F** Cell viability of Calu-3 cells over time in response to treatments. **G**–**I** Western blot analysis of protein expression within the upper chamber (Calu-3 cells). **G** Representative immunoblots for IKKβ, phosphorylated NF-κB (p-NF-κB), and Actin. **H** Relative protein expression abundance of IKKβ. **I** Relative phosphorylation abundance of *p*-NF-κB/NF-κB. Concentration of inflammatory cytokines, TNF-α (**J**) and IL-6 (**K**), in the supernatant from the upper chamber. Relative mRNA expression levels of RORγt (**L**) and IL-17A (**M**) in Jurkat T cells from the lower chamber. Concentration of TNF-α (**N**) and IL-6 (**O**) in the supernatant from the lower chamber. All data are presented as mean ± standard error of the mean (SEM). Statistical significance is indicated by: *p < 0.05, **p < 0.01, ***p < 0.001, ****p < 0.0001. *n* = 6.

PM_2.5_ reduced cell viability and TEER in a dose- and time-dependent manner. MG IIE alleviated these effects, whereas *Sphingomonas* conditioned medium (BM) alone showed no protective effect (Fig. 7A–C). MG IIE combined with BM further improved cell viability and barrier integrity, while heat-killed BM lost this effect (Fig. 7D–F).

Western blot analysis revealed that PM_2.5_ exposure significantly promoted the expression IKKβ and phosphorylation of and NF-κB of Calu-3 cell, while immunofluorescence confirmed the nuclear translocation of the p65 protein. These changes were accompanied by a significant increase in the protein expression of pro-inflammatory cytokines, TNF-α and IL-6 in upper and lower chamber supernatant, as well as the mRNA expression of the Th17 cell differentiation-related transcription factor, *rorγt* and *il-17a* in lower chamber (Fig. 7G–O). While both MG IIE and MGIIE + HK-BM alone partially inhibited these changes, the combination of MG IIE + BM was most effective. It most potently suppressed the phosphorylation of IKKβ and NF-κB and most significantly reduced the nuclear translocation of p65 and the expression of TNF-α, IL-6, and *rorγt*.

**Fig. 8:**
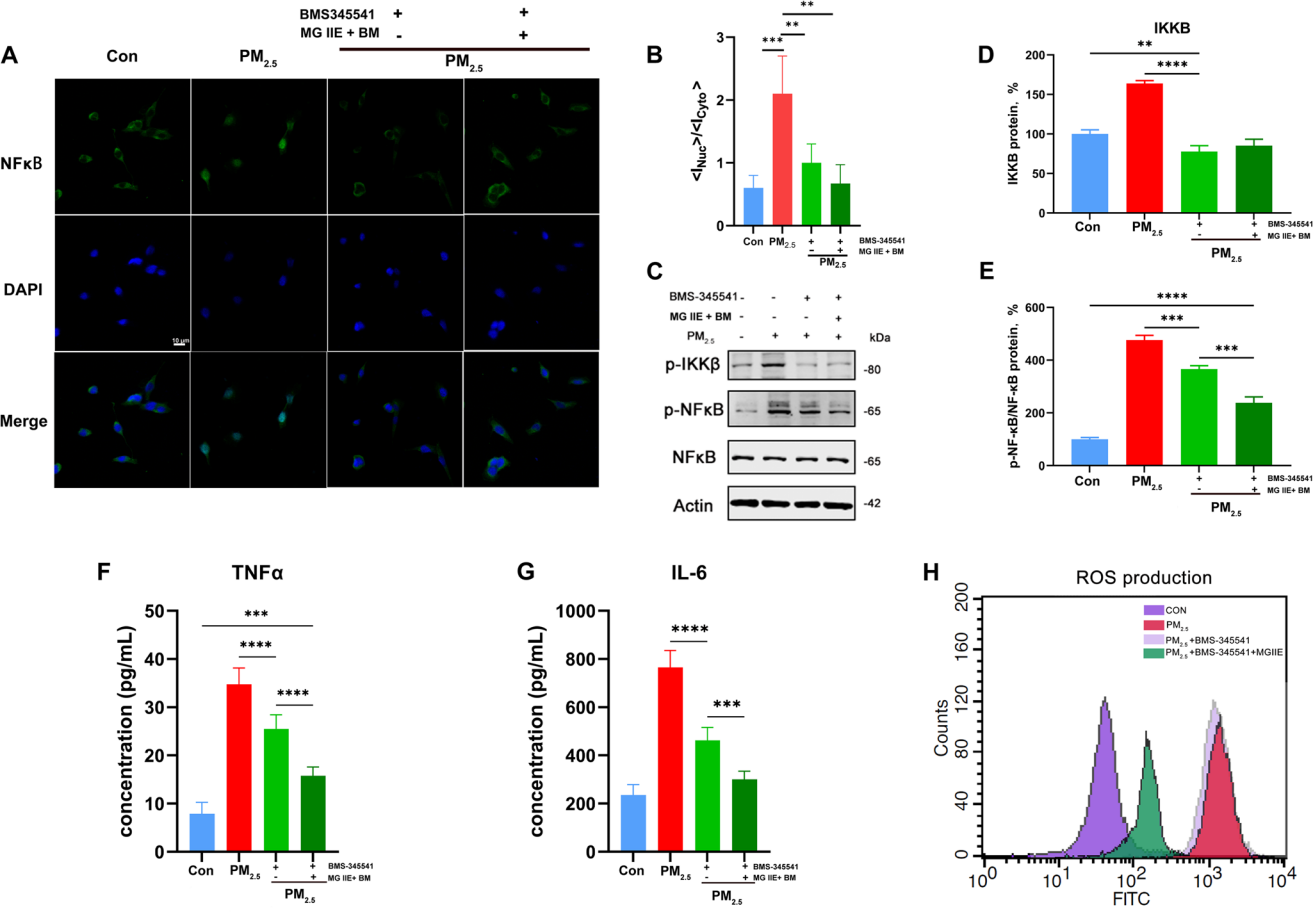
Effects of PM_2.5_, the NF-κB inhibitor BMS-345541, and MG IIE + BM on inflammatory signaling and oxidative stress in Calu-3 cells. **A** Immunofluorescence staining of Calu-3 cells showing the subcellular localization of NF-κB (green) and the nucleus (DAPI, blue), with a scale bar of 10 µm. **B** The degree of NF-κB nuclear translocation was semi-quantified by calculating the ratio of mean nuclear fluorescence intensity to mean cytoplasmic fluorescence intensity ( < I_Nuc_> / <I_Cyto_ > ) using ImageJ software, data are presented as mean ± SD (**C**) Western blot analysis of phosphorylated IKKβ (p-IKKβ), phosphorylated NF-κB (p-NF-κB), total NF-κB, and Actin as a loading control. **D**, **E** Densitometric quantification of the relative protein expression of IKKβ and the phosphorylation level of NF-κB (p-NF-κB/NF-κB). Concentrations of the pro-inflammatory cytokines TNF-α (**F**) and IL-6 (**G**) in the cell culture supernatant. **H** Flow cytometry histogram showing the level of intracellular reactive oxygen species (ROS) production. All data are presented as mean ± standard error of the mean (SEM). Statistical significance is indicated as: **p < 0.01, ***p < 0.001, ****p < 0.0001. *n* = 6.

Immunofluorescence results showed that the combined treatment with MG IIE + BM and the IKKβ inhibitor most effectively inhibited p65 protein nuclear translocation (Fig. 8A). PM_2.5_ exposure significantly increased the nuclear accumulation of NF-κB (*p* < 0.001), while the combined treatment with MG IIE + BM and the IKKβ inhibitor effectively blocked this translocation. Western blot analysis confirmed that PM_2.5_ exposure significantly increased the phosphorylation levels of both IKKβ and NF-κB. Treatment with the IKKβ inhibitor BMS-345541 significantly reduced this phosphorylation, and the addition of MG IIE further decreased these levels (Fig. 8C–E). Furthermore, enzyme-linked immunosorbent assay (ELISA) data revealed that while PM_2.5_ increased the expression of TNF-α and IL-6, the combined treatment most potently reduced their levels (Fig. 8F, G). Notably, flow cytometry analysis showed that PM_2.5_ exposure significantly increased reactive oxygen species (ROS), and while the IKKβ inhibitor did not cause a significant decrease, the inclusion of MG IIE + BM effectively reduced ROS production (Fig. 8H). These results collectively demonstrate that MG IIE + BM and the IKKβ inhibitor have a synergistic effect in mitigating the inflammatory response by modulating the NF-κB pathway and ROS production.

## Discussion

This study investigated the role of the lung microbiota in mediating the protective effects of mogroside (MG) supplementation against PM_2.5_-induced lung injury in broilers. Dietary MG supplementation study demonstrated that MG significantly alleviated pulmonary injury and reshaped the lung microbiota, with a marked increase in beneficial taxa such as *Lactobacillus* and enhanced microbial diversity. Notably, these effects were confined to the lung, as no significant changes occurred in the gut microbiota or gut-associated inflammatory markers, underscoring the lung-specific actions of MG. It is important to recognize that the pulmonary immune landscape is not isolated but heavily influenced by the gut-lung axis. Gut-resident bacteria can modulate distal lung inflammation by driving the recruitment of Th17 cells and influencing autoimmune responses^29^. Given that MG is administered orally, it likely remodels the gut microbiota^30^, which may synergistically contribute to the observed lung protection via systemic immune signaling. However, their direct presence in lung tissues confirms their ability to exert local effects^31^. Our BALF transplantation data confirms that the alone possesses sufficient immunomodulatory capacity to suppress PM_2.5_-induced inflammation, suggesting a dual mechanism involving both local and systemic pathways. Furthermore, the localized PM_2.5_ exposure model, achieved through intratracheal instillation, ensured precise dosing and minimized systemic confounding, thereby strengthening the evidence for direct pulmonary protective mechanisms of MG^32,33^.

Mechanistically, MG supplementation restored immune homeostasis by rebalancing Th17/Treg responses. PM_2.5_ exposure activated proinflammatory pathways, with TNF-α and NF-κB driving Th17 differentiation and RORγt acting as a key regulator^34,35^; MG markedly reduced the expression of these cytokines. Concomitantly, lung microbiota profiling revealed increased abundance of *Lactobacillus*, which is known for its anti-inflammatory properties and ability to promote host immune regulation^36,37^ through metabolites such as lactic acid regulation^38,39^. In contrast, the relative abundance of pathogenic *Acinetobacter*, a potent inducer of inflammatory cytokine production^40,41^, was significantly reduced. These combined microbial and immunological alterations provide a mechanistic basis for the subsequent BALF transplantation experiments designed to disentangle microbial contributions from nonmicrobial effects of MG.

BALF transplantation experiments demonstrated that BALF from MG-fed broilers (complete MG-BALF) significantly alleviated PM_2.5_-induced Lung injury, whereas its filtered counterpart (MG-BALF without microbiota) showed only modest, nonsignificant effects, and neither control BALF (normal-BALF) nor filtered control BALF (normal-BALF without microbiota) conferred protection. The pronounced reduction in Th17 activity in the complete MG-BALF group, evidenced by decreased RORγt and IL-17a expression, highlights the essential role of live microbes in mediating MG’s protective effects. Among the enriched taxa, *Sphingomonas* emerged as a key candidate, its reported ability to metabolize saponins and generate bioactive derivatives that can suppress Th17 responses^42,43^. Its consistently high abundance in MG-derived BALF, irrespective of filtration, suggests a selective interaction with MG and potential metabolic compatibility. In contrast, Lactobacillus, which was enriched in the first experiment, showed limited changes in the second, and its contribution to Th17 modulation appeared minor under the short transplantation regimen^44,45^. These results underscore that the protective effects of MG are closely tied to specific microbial partners—particularly *Sphingomonas*—and their metabolites, which likely orchestrate the suppression of pulmonary inflammation.

Metabolomic profiling revealed that taurine, lactate, galactose, tryptophan, and sphinganine were significantly altered by MG-BALF transplantation, with KEGG analysis highlighting enrichment in taurine, galactose, sphinganine, tryptophan, and membrane metabolism pathways. Notably, taurine and lactate were upregulated in the MG-BALF group, suggesting a pivotal role in mediating the anti-inflammatory effects of MG. Elevated levels of taurine and lactate are particularly relevant, as both metabolites are known to enhance immune regulation and mitigate oxidative stress, consistent with their observed association with reduced inflammation^46–48^. Joint microbiome–metabolome analysis indicated that *Sphingomonas* was positively correlated with taurine and lactate, whereas *Acidithiobacillus*, enriched in PM_2.5_-exposed broilers, showed negative correlations, implying its contribution to a proinflammatory metabolic profile. In contrast, *Lactobacillus* exhibited limited associations with these metabolites, suggesting its role in MG-mediated protection may act primarily through immune modulation rather than direct metabolic regulation. These findings were substantiated in vivo and in vitro. In dietary MG supplementation study, MG supplementation significantly elevated hepatic taurine, whereas in BALF transplantation study, MG-BALF donors retained higher levels of taurine and MG IIE compared with controls. Importantly, in the BALF of recipients, complete MG-BALF transplantation resulted in significantly decreased MG IIE but increased mogrol levels relative to filtered MG-BALF recipients, directly linking microbial activity to MG metabolism in vivo. Complementary in vitro assays further demonstrated that *Sphingomonas* efficiently degraded MG IIE, with concurrent β-glucosidase induction, medium acidification, and the time-dependent conversion of MG IIE into mogrol. These results support the notion that MG IIE, upon reaching the alveolar space—especially under conditions of PM₂.₅-induced epithelial barrier disruption—can serve as a microbial substrate, leading to the generation of bioactive aglycones and acids, such as mogrol and potentially taurine, that may contribute to local anti-inflammatory effects. Together, these data suggest a novel respiratory axis of phytochemical-microbiota interaction that warrants further mechanistic investigation. This result further support the hypothesis that MG IIE exerts its protective effect against PM₂.₅-induced lung injury at least partially via lung microbiota-mediated biotransformation.

The results of in vitro co-culture mechanistic validation further validated the mechanistic basis underlying the protective effects of MG and MG-associated microbes. In vivo, BALF transplantation highlighted the essential contribution of live microbial communities—particularly *Sphingomonas*—in shaping the pulmonary metabolite landscape and alleviating PM_2.5_-induced inflammation. Metabolomic and degradation analyses demonstrated that *Sphingomonas* hydrolyzed MG IIE into mogrol and modulated key metabolites, such as taurine and lactate, which are known to regulate oxidative stress and immune balance. Extending these findings to the in vitro Transwell co-culture model, we demonstrated that MG IIE alone partially protected epithelial integrity, whereas the addition of *Sphingomonas*-derived metabolites synergistically enhanced this effect, reducing ROS levels and restoring barrier function. Mechanistically, this protection was associated with the suppression of IKKβ phosphorylation, NF-κB activation, and NF-κB nuclear translocation, leading to decreased secretion of TNF-α and IL-6 in the epithelial compartment and reduced expression of Th17-associated markers (RORγt, IL-17a) in the lymphocyte compartment. Importantly, the synergistic action of MG IIE and bacterial metabolites was superior to either treatment alone, emphasizing the necessity of microbe–mogroside interactions in optimizing anti-inflammatory outcomes. The absence of Th17 differentiation data in the lower chamber of the results of in vitro co-culture mechanistic validation is likely attributable to the inhibition of NF-κB in the upper chamber, which resulted in pro-inflammatory cytokine concentrations that were diluted to levels insufficient to trigger Th17 differentiation upon reaching the lower chamber. These results bridge the in vivo and in vitro observations, establishing that MG supplementation enhances pulmonary resilience against PM_2.5_ not only by altering microbial composition but also by enabling specific microbial metabolism of MG that directly modulate NF-κB–Th17 signaling.

It is crucial to interpret these findings within the context of avian comparative physiology. Unlike the tidal respiration and alveolar structure of mammals, the avian respiratory system features unidirectional airflow and rigid parabronchi coupled with distinct air sacs. These air sacs, which lack efficient mucociliary clearance mechanisms, serve as reservoirs that facilitate the accumulation and sedimentation of fine particles like PM_2.5_, making the avian lung an ‘anatomical trap’ for airborne pollutants. Additionally, the thinner blood-gas barrier in birds—evolved for high-efficiency gas exchange—renders the tissue more susceptible to oxidative stress and inflammatory translocation compared to mammals. Consequently, while the broiler serves as a highly sensitive ‘sentinel model’ for elucidating the molecular mechanisms of PM_2.5_ toxicity (NF-κB activation), the structural absence of alveoli necessitates caution when directly translating specific histological lesions to human respiratory pathology. An ASV-based workflow (DADA2/Deblur) may provide higher resolution; however, our main conclusions are supported by large genus-level shifts and independent functional validation, and are therefore unlikely to be altered by switching from OTUs to ASVs. Notwithstanding the insights gained, there remains ample scope for expanding our comprehension of the pulmonary microenvironment. Our current analysis was restricted to key bacterial genera; however, the lung microbiome represents a multifaceted ecosystem whose holistic interactions require more granular exploration. Moreover, the detailed molecular dialogue, specifically how these microbial constituents modulate host immunity, remains to be deciphered. It is also important to acknowledge that while the avian model provides high sensitivity to airborne pollutants, species-specific respiratory mechanics necessitate caution in translational interpretation. Future endeavors should therefore aim to profile the global microbial ecology and longitudinally assess the sustained impact of MG on pulmonary homeostasis.

In summary, this study demonstrates that mogroside exerts protective effects against PM_2.5_-induced pulmonary inflammation through a lung microbiota–dependent mechanism. Specifically, *Sphingomonas*-mediated biotransformation of MG IIE generates bioactive metabolites, including mogrol and taurine which collectively preserve epithelial barrier integrity, modulate NF-κB activation, and suppress Th17 overactivation. BALF transplantation experiments confirmed the essential role of live lung microbiota in mediating these effects, while in vitro Transwell co-culture studies validated the mechanistic link between microbial metabolites and NF-κB–Th17 signaling. These findings reveal a novel microbiota–metabolite–immune axis in the lung and highlight the therapeutic potential of targeting lung microbiota–mediated mogroside metabolism to mitigate PM_2.5_–induced lung injury.

## Supplementary information

Supplementary Materials

## Data Availability

The raw 16S rRNA gene sequencing data are available at the NCBI Sequence Read Archive (SRA), under BioProject (PRJNA1209218, PRJNA1210855).

## References

[1] Wang, K., Shen, D., Dai, P. & Li, C. Particulate matter in poultry house on poultry respiratory disease: a systematic review. *Poultry Sci.* **102**, 102556 (2023). 36848758 10.1016/j.psj.2023.102556 PMC9982681

[2] Yang, D. et al. Antimicrobial resistance in China across human, animal, and environment sectors – a review of policy documents using a governance framework. *Lancet Regional Health – Western Pacific* **48**, 101111 (2024). 38948912 10.1016/j.lanwpc.2024.101111 PMC11214315

[3] Hu, Y. J. & Cowling, B. J. Reducing antibiotic use in livestock, China. *B World Health Organ* **98**, 360 (2020). 10.2471/BLT.19.243501 PMC7265937 32514201

[4] Shen, D. et al. Inflammation-associated pulmonary microbiome and metabolome changes in broilers exposed to particulate matter in broiler houses. *J. Hazard Mater.* **421**, 126710 (2022). 34332479 10.1016/j.jhazmat.2021.126710

[5] Dai, P., Shen, D., Tang, Q., Huang, K. & Li, C. PM(2.5) from a broiler breeding production system: The characteristics and microbial community analysis. *Environ. Pollut.* **256**, 113368 (2020). 31676097 10.1016/j.envpol.2019.113368

[6] Zhao, C. N. et al. Emerging role of air pollution in autoimmune diseases. *Autoimmun. Rev.* **18**, 607 (2019). 30959217 10.1016/j.autrev.2018.12.010

[7] Yu, Z. X. et al. The ratio of Th17/Treg cells as a risk indicator in early acute respiratory distress syndrome. *Crit. Care* **19**, 82 (2015). 25887535 10.1186/s13054-015-0811-2 PMC4355972

[8] Thomas, R., Qiao, S. & Yang, X. Th17/Treg Imbalance: Implications in Lung Inflammatory Diseases. *Int. J. Mol. Sci.* **24**, 4865 (2023). 36902294 10.3390/ijms24054865 PMC10003150

[9] Ma, R., Su, H., Jiao, K. & Liu, J. Role of Th17 cells, Treg cells, and Th17/Treg imbalance in immune homeostasis disorders in patients with chronic obstructive pulmonary disease. *Immun. Inflamm. Dis.* **11**, e784 (2023). 36840492 10.1002/iid3.784 PMC9950879

[10] Schnell, J. T. et al. The ‘T(reg) paradox’ in inflammatory arthritis. *Nat. Rev. Rheumatol.* **21**, 9 (2025). 39653758 10.1038/s41584-024-01190-w

[11] Yero, A., Bouassa, R. M., Ancuta, P., Estaquier, J. & Jenabian, M. A. Immuno-metabolic control of the balance between Th17-polarized and regulatory T-cells during HIV infection. *Cytokine Growth F. R.* **69**, 1 (2023). 10.1016/j.cytogfr.2023.01.001 36681548

[12] Yang, Y., Li, S. & Xu, H. BPIFA1 alleviates allergic rhinitis by regulating the NF-kappaB signaling pathway and Treg/Th17 balance. *Int J. Rheum. Dis.* **27**, e15372 (2024). 39450979 10.1111/1756-185X.15372

[13] Guo, Q. et al. Recent Advances in the Distribution, Chemical Composition, Health Benefits, and Application of the Fruit of Siraitia grosvenorii. *Foods* **13**, 2278 (2024). 39063362 10.3390/foods13142278 PMC11275593

[14] Benucci, I., Lombardelli, C. & Esti, M. A comprehensive review on natural sweeteners: impact on sensory properties, food structure, and new frontiers for their application. *Crit. Rev. Food Sci.* **65**, 4615–4633 (2025). 10.1080/10408398.2024.2393204 39154209

[15] Shivani et al. Introduction, adaptation and characterization of monk fruit (Siraitia grosvenorii): a non-caloric new natural sweetener. *Sci. Rep.-UK* **11**, 6205 (2021). 10.1038/s41598-021-85689-2 PMC7973523 33737610

[16] Wei, Y. et al. Network pharmacology and experimental verification to explore the anti-inflammatory activities of triterpenoids from Siraitia grosvenorii. *Nat. Prod. Res.* **1** (2024). 10.1080/14786419.2024.2412312 39381933

[17] Liu, H. et al. Anti-depression-like effect of Mogroside V is related to the inhibition of inflammatory and oxidative stress pathways. *Eur. J. Pharm.* **955**, 175828 (2023). 10.1016/j.ejphar.2023.175828 37364672

[18] Mo, Q. et al. Protective Effects of Mogroside V on Oxidative Stress Induced by H(2)O(2) in Skin Fibroblasts. *Drug Des. Devel Ther.* **15**, 4901 (2021). 34880600 10.2147/DDDT.S337524 PMC8647757

[19] Li, Y. et al. Mogroside V inhibits LPS-induced COX-2 expression/ROS production and overexpression of HO-1 by blocking phosphorylation of AKT1 in RAW264.7 cells. *Acta Bioch Bioph Sin.* **51**, 365 (2019). 10.1093/abbs/gmz014 30877761

[20] Wang, L. et al. Cucurbitane glycosides derived from mogroside IIE: structure-taste relationships, antioxidant activity, and acute toxicity. *Molecules (Basel, Switz.)* **19**, 12676 (2014). 10.3390/molecules190812676 PMC6271920 25140446

[21] Cui, S. et al. Post-Ripening and Key Glycosyltransferase Catalysis to Promote Sweet Mogrosides Accumulation of Siraitia grosvenorii Fruits. *Molecules (Basel, Switz.)* **28**, 4697 (2023). 10.3390/molecules28124697 PMC10303746 37375251

[22] Lü, W., Ren, G., Shimizu, K., Li, R. & Zhang, C. Mogroside ⅡE, an in vivo metabolite of sweet agent, alleviates acute lung injury via Pla2g2a-EGFR inhibition. *Food Sci. Hum. Well* **13**, 299 (2024).

[23] Kuziel, G. A. et al. Functional diversification of dietary plant small molecules by the gut microbiome. *Cell* **188**, 1967 (2025). 40056901 10.1016/j.cell.2025.01.045 PMC12671244

[24] King, A. Exploring the lung microbiome’s role in disease. *Nature*. 10.1038/d41586-024-01123-3 (2024). 10.1038/d41586-024-01123-3 38632423

[25] Yi, X., Gao, J. & Wang, Z. The human lung microbiome-A hidden link between microbes and human health and diseases. *Imeta* **1**, e33 (2022). 38868714 10.1002/imt2.33 PMC10989958

[26] Liu, B. et al. Characterizing microbiota and metabolomics analysis to identify candidate biomarkers in lung cancer. *Front Oncol.* **12**,1058436 (2022). 10.3389/fonc.2022.1058436 PMC9705781 36457513

[27] Yan, Z. et al. Multi-omics analyses of airway host-microbe interactions in chronic obstructive pulmonary disease identify potential therapeutic interventions. *Nat. Microbiol.* **7**, 1361 (2022). 35995842 10.1038/s41564-022-01196-8

[28] Li, Y. et al. Mogroside V protects Lipopolysaccharides-induced lung inflammation chicken via suppressing inflammation mediated by the Th17 through the gut-lung axis. *J. Anim. Sci.* **103**, skae388 (2024). 10.1093/jas/skae388 PMC11773191 39716346

[29] Bradley, C. P. et al. Segmented Filamentous Bacteria Provoke Lung Autoimmunity by Inducing Gut-Lung Axis Th17 Cells Expressing Dual TCRs. *Cell Host Microbe* **22**, 697 (2017). 29120746 10.1016/j.chom.2017.10.007 PMC5749641

[30] Xiao, R. et al. Modulation of Gut Microbiota Composition and Short-Chain Fatty Acid Synthesis by Mogroside V in an In Vitro Incubation System. *ACS Omega* **6**, 25486 (2021). 34632206 10.1021/acsomega.1c03485 PMC8495861

[31] Xu, F. et al. Exploring in vitro, in vivo metabolism of mogroside V and distribution of its metabolites in rats by HPLC-ESI-IT-TOF-MS(n). *J. Pharm. Biomed.* **115**, 418 (2015). 10.1016/j.jpba.2015.07.024 26280925

[32] Budden, K. F. et al. Emerging pathogenic links between microbiota and the gut-lung axis. *Nat. Rev. Microbiol* **15**, 55 (2017). 27694885 10.1038/nrmicro.2016.142

[33] Wang, L. et al. The Bidirectional Gut-Lung Axis in Chronic Obstructive Pulmonary Disease. *Am. j. Resp. Crit. Care* **207**, 1145 (2023). 10.1164/rccm.202206-1066TR PMC10161745 36883945

[34] Jie, X. L. et al. Pi-Pa-Run-Fei-Tang alleviates lung injury by modulating IL-6/JAK2/STAT3/IL-17 and PI3K/AKT/NF-kappaB signaling pathway and balancing Th17 and Treg in murine model of OVA-induced asthma. *J. Ethnopharmacol.* **317**, 116719 (2023). 37268260 10.1016/j.jep.2023.116719

[35] Aso, K. et al. Itaconate ameliorates autoimmunity by modulating T cell imbalance via metabolic and epigenetic reprogramming. *Nat. Commun.* **14**, 984 (2023). 36849508 10.1038/s41467-023-36594-x PMC9970976

[36] Glick, V. J. et al. Vaginal lactobacilli produce anti-inflammatory beta-carboline compounds. *Cell Host Microbe* **32**, 1897 (2024). 39423813 10.1016/j.chom.2024.09.014 PMC11694765

[37] Matuchansky, C. Anti-inflammatory lactobacilli: strain specificity. *GUT* **61**, 786 (2012). 784. 21846781 10.1136/gutjnl-2011-300881

[38] Nicola, T. et al. A lactobacilli-based inhaled live biotherapeutic product attenuates pulmonary neutrophilic inflammation. *Nat. Commun.* **15**, 7113 (2024). 39160214 10.1038/s41467-024-51169-0 PMC11333600

[39] Fangous, M. S. et al. Lactobacilli intra-tracheal administration protects from Pseudomonas aeruginosa pulmonary infection in mice - a proof of concept. *Benef. Microbes* **10**, 893 (2019). 31965833 10.3920/BM2019.0069

[40] Palmer, L. D. et al. Dietary zinc deficiency promotes Acinetobacter baumannii lung infection via IL-13 in mice. *Nat. Microbiol* **9**, 3196 (2024). 39548344 10.1038/s41564-024-01849-w PMC11800279

[41] Liu, Z. & Xu, W. Neutrophil and Macrophage Response in Acinetobacter Baumannii Infection and Their Relationship to Lung Injury. *Front Cell Infect. Mi* **12**, 890511 (2022). 10.3389/fcimb.2022.890511 PMC9298752 35873147

[42] Lin, D. et al. The niche-specialist and age-related oral microbial ecosystem: crosstalk with host immune cells in homeostasis. *Microb. Genomics* **8**, 000811 (2022). 10.1099/mgen.0.000811 PMC9455711 35731208

[43] Asaf, S., Numan, M., Khan, A. L. & Al-Harrasi, A. Sphingomonas: from diversity and genomics to functional role in environmental remediation and plant growth. *Crit. Rev. Biotechnol.* **40**, 138 (2020). 31906737 10.1080/07388551.2019.1709793

[44] Zhu, Y. et al. Lactobacillus murinus alleviated lung inflammation induced by PAHs in mice. *Ecotox Environ Safe* **281**, 116662 (2024). 10.1016/j.ecoenv.2024.116662 38944008

[45] Bernard-Raichon, L. et al. A Pulmonary Lactobacillus murinus Strain Induces Th17 and RORgammat(+) Regulatory T Cells and Reduces Lung Inflammation in Tuberculosis. *J. Immunol.* **207**, 1857 (2021). 34479945 10.4049/jimmunol.2001044

[46] Ping, Y. et al. Taurine enhances the antitumor efficacy of PD-1 antibody by boosting CD8(+) T cell function. *Cancer Immunol. Immun.* **72**, 1015 (2023). 10.1007/s00262-022-03308-z PMC10991389 36261540

[47] Chen, J. et al. Protective effect of taurine on sepsis‑induced lung injury via inhibiting the p38/MAPK signaling pathway. *Mol. Med. Rep.* **24**, 653 (2021). 34278479 10.3892/mmr.2021.12292 PMC8299207

[48] Colbert, L. E. et al. Tumor-resident Lactobacillus iners confer chemoradiation resistance through lactate-induced metabolic rewiring. *Cancer cell* **41**, 1945 (2023). 37863066 10.1016/j.ccell.2023.09.012 PMC10841640

