# Supplementary material for: Lung microbiota-mediated biotransformation of mogroside preserves pulmonary barrier integrity and attenuates PM2.5-induced inflammation via NF-κB–Th17 modulations
Source: NPJ Biofilms Microbiomes. 2026 May 5;12:133. doi: 10.1038/s41522-026-00992-y (PMC13350695; doi:10.1038/s41522-026-00992-y)
Supplement: Supplementary file 1 — Supplementary Materials [file 41522_2026_992_MOESM1_ESM.pdf]

Collection of fine particulate matter ($PM_{2.5}$)

The $PM_{2.5}$ used for tracheal instillation experiments was collected directly from a commercial broiler house to replicate the actual inhalation conditions experienced by broilers. The broiler house, oriented north-south with dimensions of 90 m × 11 m, operated as a cage-free system using rice hull bedding, a common practice in broiler farming across China. During sampling, the filters were placed in the $PM_{2.5}$ impactor head of eagle 2030 medium-flow environmental air particle sampler for $PM_{2.5}$ collection. The particle sampler, equipped with the $PM_{2.5}$ impactor head, was positioned at the center of the poultry house at a height of 1 meter, with a sampling flow rate of 100 L/min for 23 hours each day. Post-sampling, the filters loaded with $PM_{2.5}$ were returned to the membrane boxes, sealed with parafilm, and stored at -20°C. The $PM_{2.5}$-laden filters were cut into small pieces using plastic scissors and placed in beakers containing 50 mL of ultrapure water. The mixture was subjected to ultrasonic agitation at 80 Hz for 30 minutes. The $PM_{2.5}$ suspension was then filtered through six layers of gauze into a conical flask and subsequently aliquoted into 2 mL centrifuge tubes. The samples were centrifuged at 12,000 rpm and 4°C for 40 minutes. The supernatant was discarded, and approximately 0.5 mL of the remaining liquid was pooled from 2-3 tubes and centrifuged again, a process repeated three times. After the final centrifugation, the remaining liquid (~0.5 mL) was freeze-dried using a lyophilizer and further dried in a desiccator for 6 hours before weighing. The dried $PM_{2.5}$ was dissolved in a small amount of 0.75% saline solution, transferred to a new sterile container, and the centrifuge tubes were weighed again. The $PM_{2.5}$ mass was determined by the difference in the weights of the centrifuge tubes before and after $PM_{2.5}$ collection. Finally, the $PM_{2.5}$ samples were resuspended in 0.75% saline solution to a

concentration of 2 mg/mL and stored at 4°C for subsequent experiments.

Tracheal instillation of $PM_{2.5}$ in broiler chickens

The method for preparing a $PM_{2.5}$-induced pneumonia animal model in chickens and evaluating the model. First, preoperative preparation includes sterilizing the gavage needle with a high-pressure autoclave, collecting and lyophilizing $PM_{2.5}$ particles, then preparing a $PM_{2.5}$ suspension to ensure uniform mixing. A clean workspace is set up, and a chicken anesthesia chamber is prepared with ether. Next, 500 µl of the $PM_{2.5}$ suspension is drawn into a 1 mL syringe for use during the procedure. The chicken is placed in the anesthesia chamber, and after monitoring anesthesia depth for about 20 seconds, the chicken is anesthetized. For the tracheal intubation, the anesthetized chicken is gently positioned on the operating table, securing the wings with Velcro to keep the airway open. The syringe is tilted, and the gavage needle with the $PM_{2.5}$ suspension is inserted along the natural path of the trachea, allowing the particles to be inhaled into the alveoli with each breath. After the procedure, the chicken is monitored closely in a warm, dry area, ensuring the $PM_{2.5}$ suspension reaches the alveoli, and the chicken is returned to the cage for normal feeding once it regains consciousness, with daily observation and weight measurements. Finally, after seven days of continuous infusion, the chicken is euthanized by asphyxiation with dry ice. The concentration and injection amount of $PM_{2.5}$ are obtained through calculation formulas based on our measured daily respiration rate of chickens, the measured concentration in commercial chicken houses, and the actual concentration in exposure tests. Refer to Table S1.

Aseptic Collection of Bronchoalveolar Lavage Fluid (BALF)

Pre-cooled, sterile 0.75% saline solution was meticulously prepared and reserved under

aseptic conditions for the BALF procedure. A sterile 30 mL syringe equipped with a sterile, disposable soft tube served as the lavage device. Chickens were anesthetized using an approved protocol, ensuring minimal stress and humane handling. The neck region was disinfected with 70% ethanol before carefully dissecting the skin and muscles to expose the trachea. A small incision was made in the trachea using a sterile scalpel. Throughout the process, sterile gloves, instruments, and materials were used to prevent contamination. The sterile soft tube, preloaded with 30 mL of sterile saline, was gently inserted into the trachea. The interface between the tube and trachea was secured using a sterile fine thread to maintain a closed system. Saline was slowly and evenly injected into the lungs, ensuring proper distribution. The lavage process involved gently aspirating the saline back into the syringe, repeating the injection and aspiration cycle three times to collect sufficient BALF. The collected BALF was immediately transferred to sterile centrifuge tubes, which were pre-labeled and stored on ice. To ensure thorough sampling, an additional 30 mL of sterile saline was drawn, and the lavage procedure was repeated once under identical sterile conditions. Both the control group and the broilers fed with mogrosides were healthy, with minimal immune cells observed in their BALF. To ensure greater rigor in our analysis, we performed a simple removal of immune cells from the BALF. This was achieved through low-speed centrifugation (400 ×g, 10 minutes) to eliminate cellular debris and mucus, after which the supernatant was retained and stored at 4°C for subsequent use. This centrifugation speed and time does not precipitate or separate microorganisms, ensuring their retention in the supernatant for further analysis. Strict adherence to sterile techniques throughout the procedure ensured the integrity of the BALF samples for downstream analyses.

16S rRNA genes sequencing and analysis

Total genomic DNA was extracted using the sodium dodecyl sulphate (SDS) method (Sambrook and Russell, 2001), and its concentration and purity were verified with 1% agarose gel electrophoresis and a NanoDrop 2000 spectrophotometer. The DNA was diluted to a concentration of 1 ng·μL^{-1} using sterile water. For amplicon sequencing, the V4 hypervariable region of the 16S rRNA gene was amplified using primers 515F and 806R with barcodes. The PCR reactions included Phusion® High-Fidelity PCR Master Mix, primers, and approximately 10 ng of template DNA, following a thermal cycling protocol involving denaturation, annealing, elongation, and final extension steps. The resulting PCR products were purified using the GeneJET™ Gel Extraction Kit. Sequencing libraries were prepared with the Ion Plus Fragment Library Kit and assessed for quality using a Qubit® 2.0 Fluorometer before sequencing on the Ion S5™ XL platform to generate 400–600 bp single-end reads. For data processing, single-end reads were demultiplexed based on barcodes, and low-quality reads, along with chimera sequences, were filtered out using Cutadapt (v1.9.1) and the UCHIME algorithm. Operational taxonomic units (OTUs) were clustered at 97% similarity, and abundance data were normalized to the sample with the fewest sequences. The α diversity indices, including observed species, ACE, Chao1, Shannon, and Simpson indices, were calculated with QIIME (v1.9.1) to assess species diversity within samples, while β diversity was analyzed using principal coordinates analysis (PCoA), partial least squares discriminant analysis (PLS-DA), and hierarchical clustering based on Bray-Curtis distances. To identify bacterial taxa contributing to intergroup differences, linear discriminant analysis (LDA) effect size (LEfSe) was performed. Functional predictions based on the 16S rRNA OTU data were conducted using Tax4Fun, with functional

categorization aligned to the Kyoto Encyclopedia of Genes and Genomes (KEGG) Orthology. Visualization of functional predictions was done using STAMP (v2.1.0), while other figures were created with R software (v3.4.3) or QIIME (v1.9.1).

Metabolomic Analysis Workflow and Data Processing

For metabolomic analysis, six lung tissue samples from the groups were randomly selected. Lung tissues (100 mg) were ground in liquid nitrogen, resuspended in prechilled 80% methanol with 0.1% formic acid, and vortexed thoroughly. After incubating on ice for 5 minutes, samples were centrifuged at 15,000 rpm at 4°C for 5 minutes, and the supernatant was diluted to a final concentration of 60% methanol with LC-MS-grade water. The solution was filtered through a 0.22 μm filter, centrifuged again, and the resulting supernatant was subjected to LC-MS analysis. Quality control (QC) samples were prepared by pooling equal volumes from each experimental sample, while blank samples consisted of 60% methanol with 0.1% formic acid. The metabolite quantification was carried out using a Vanquish UHPLC system coupled with an Orbitrap Q Exactive mass spectrometer. Lung samples were injected into a Hyperil Gold column (100 mm × 2.1 mm, 1.9 μm) and analyzed over a 16-minute linear gradient with a flow rate of 0.2 mL/min. Positive and negative ionization modes employed different mobile phase combinations, with solvent gradients adjusted accordingly. The mass spectrometer operated in a scanning range of 70–1050 m/z, with optimized parameters including a spray voltage of 3.2 kV, capillary temperature of 320°C, sheath gas flow rate of 35 arb, and aux gas flow rate of 10 arb. Raw UHPLC-MS/MS data were processed using Compound Discoverer 3.0 (Thermo Fisher), involving peak alignment, peak picking, and quantitation under stringent conditions

(e.g., retention time tolerance of 0.2 min, mass tolerance of 5 ppm, and minimum intensity of 100,000). Normalized data were used for molecular formula predictions, and metabolite identification was achieved through mzCloud and ChemSpider database matching. Statistical analyses were performed using R, Python, and CentOS. For non-normally distributed data, area normalization was applied. Metabolites were annotated using the KEGG. Visualization tools, including volcano plots and heatmaps (generated via the Pheatmap package in R), were employed to highlight significant metabolites and their clustering patterns. Pearson correlation analysis was also conducted to examine relationships between metabolites, with statistical significance set at $p < 0.05$. Functional and pathway enrichment analyses were performed using the KEGG database, where pathways were deemed enriched if $x/n > y/N$ and statistically significant when $p < 0.05$. Receiver operating characteristic (ROC) curves were constructed to evaluate potential biomarker performance, with AUC values > 0.9 indicating high predictive accuracy.

Cell Culture and Experimental Design

Calu-3 human bronchial epithelial cells (ATCC® HTB-55™, Manassas, VA, USA) were cultured in DMEM (Gibco, Thermo Fisher Scientific) supplemented with 10% FBS and 1% penicillin–streptomycin. Jurkat T lymphocytes (ATCC® TIB-152™) were maintained in RPMI-1640 (Gibco) with 10% FBS. For co-culture, Calu-3 cells were seeded onto 12-well Transwell inserts (0.4 μm pore, Corning) at 1×10^5 cells/cm^2 and cultured for 8 days to form a polarized barrier, confirmed by TEER using an EVOM2 voltohmmeter (World Precision Instruments, Sarasota, FL, USA). Jurkat cells were seeded in the lower chamber at 5×10^5 cells/well. In Phase 1, Calu-3 monolayers were exposed apically to PM2.5 (25–100 μg/mL),

bacterial metabolites (BM, 1–5% v/v), or MG IIE (25–100 μg/mL) for 24 h, and cell viability was assessed via CCK-8 assay (Dojindo, Japan) to determine optimal concentrations. In Phase 2, mature monolayers were challenged with $PM_{2.5}$ or $PM_{2.5}$ + BM (live or heat-inactivated) in the apical chamber, while MG IIE was added basolaterally; TEER was monitored at multiple time points to evaluate barrier integrity.

Phase 3 investigated NF-κB–Th17 signaling using experimental groups: Control, $PM_{2.5}$, $PM_{2.5}$ + NF-κB inhibitor (BMS-345541, 10 μM; Selleck Chemicals, Houston, TX, USA), and $PM_{2.5}$ + NF-κB inhibitor + MG IIE + BM. Total and phosphorylated NF-κB and IKKβ were measured by Western blot (Cell Signaling Technology, Danvers, MA, USA), and NF-κB nuclear translocation was examined by confocal immunofluorescence (Leica TCS SP8, Leica Microsystems, Wetzlar, Germany). Supernatants were collected for TNF-α and IL-6 ELISA (R&D Systems, Minneapolis, MN, USA), and Jurkat cells were analyzed for RORγt and IL-17a expression via RT-qPCR (Applied Biosystems, Thermo Fisher Scientific). ROS levels were quantified by DCFDA staining and flow cytometry (BD FACSCanto II, BD Biosciences, San Jose, CA, USA). All treatments were performed in triplicate, with six independent repeats, and data were analyzed using GraphPad Prism 9 (GraphPad Software, San Diego, CA, USA).

Table S1 Indoor simulation of commercial chicken house $PM_{2.5}$ concentration

	Broiler house	Experiment chambers		
		control	4 mg·m^{-3}	8 mg·m^{-3}
$PM_{2.5}$	0.713 ± 0.021	0.256 ± 0.052	1.264 ± 0.120	1.976 ± 0.083

The $PM_{2.5}$ concentration (per kilogram) = tidal volume × respiratory rate × time × actual $PM_{2.5}$ concentration in the chicken house. $PM_{2.5}$ concentration (per kilogram) = 13.7 ml × 54.4 breaths/min × 60 min × 24 h × (1 mg/m^3 × 10^{-6}) mg/mL ≈ 1 mg/d.

Table S2 Primers used for quantitative real-time PCR

Gene	Sequence (5'to 3')	Size (bp)
β-actin	F: TTGTTGACAATGGCTCCGGT<br>R: TCTGGGCTTCATCACCAACG	83
Muc1	F: ACGCCTTCTTCAGCAGCAACTC<br>R: AGCAGCAGATGTGAGCAGTGATG	198
Tnf-α	F: GGCGTGGTGCTGAGAAGG<br>R: CTCGGAGAAGCGGCTGAC	149
Tgf-β	F: ATGTGTTCCGCTTTAACGTGTC<br>R: GCTGCTTTGCTATATGCTCATC	193
Il-1β	F: CCGAGGAGCAGGGACTTTG<br>R: AAGGACTGTGAGCGGGTGTAG	120
Ahr	F: CACCTACGCCAGTCGCAAGC<br>R: CCTGTGCCTCTTGGATGGATTGG	172
Foxp3	F: AACGGCGAGACACCTTC<br>R: TTCGGAGACTTTAATCCACTA	158
Rorγt	F: CACCCCCAGCTTCACCATAG<br>R: GCAGCTCAATCTCCAATGCG	169
Il-17a	F: AAGGTGATACGGCCAGGACT<br>R: GAGTTCACGCACCTGGAATG	128
Il-17f	F: GCCCTACATCAGGAATCGCA<br>R: AGTTCAAGCAGCCCAAGAGG	102
Il-22	F: CCTTAACCCTGAGATCCCGTAGA<br>R: CCACATCCTCAGCATACGGG	137

Figure S1 Chemical structures of mogroside

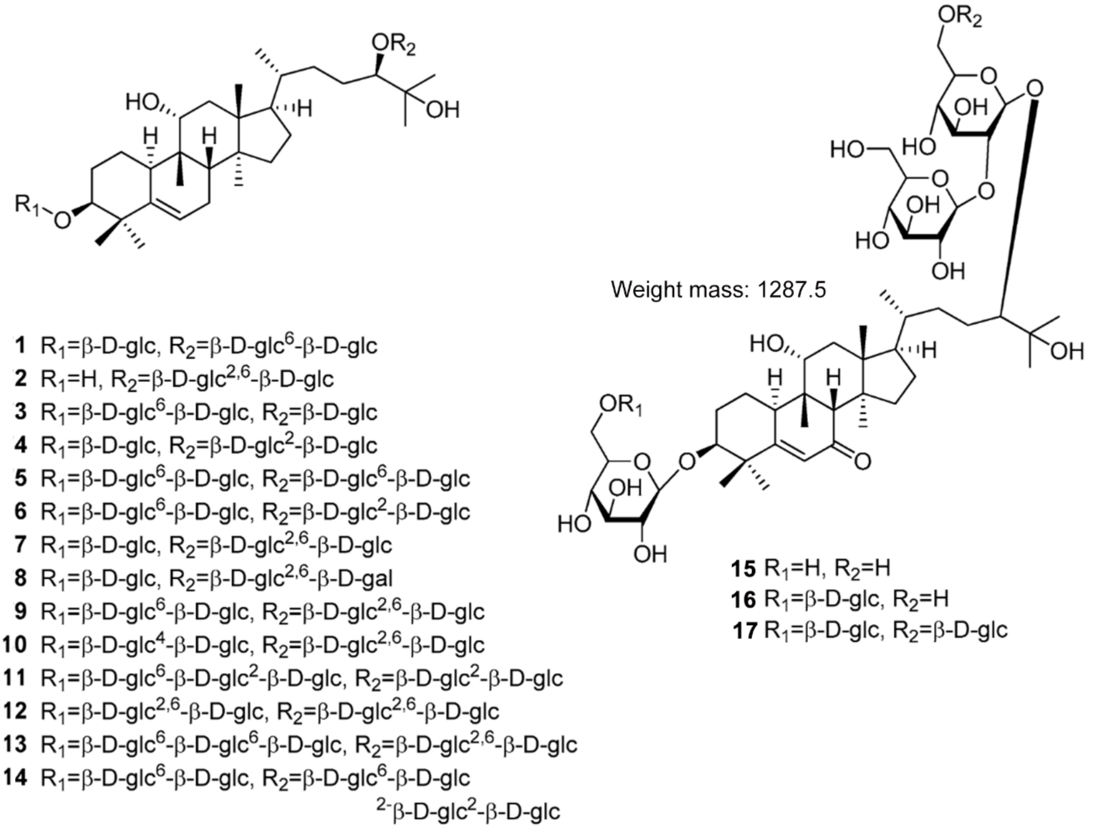

Mogrosides are cucurbitane-type triterpenoid glycosides derived from Siraitia grosvenorii (monk fruit), known for their intense sweetness. Mogroside V, the major sweet component, contains five β-D-glucose units—two at C-3 (as a disaccharide) and three at C-24 (as a branched trisaccharide)—and exhibits a molecular weight of 1287.5. The substitution degree is 5, with sugars linked via (1→2) and (1→6) glycosidic bonds. The presence of an 11α-hydroxy group and specific glycosylation patterns contribute to its sweet taste and high relative sweetness (RS = 425).

Figure S2 Mogroside on intestinal tissue of broilers with pneumonia induced by $PM_{2.5}$

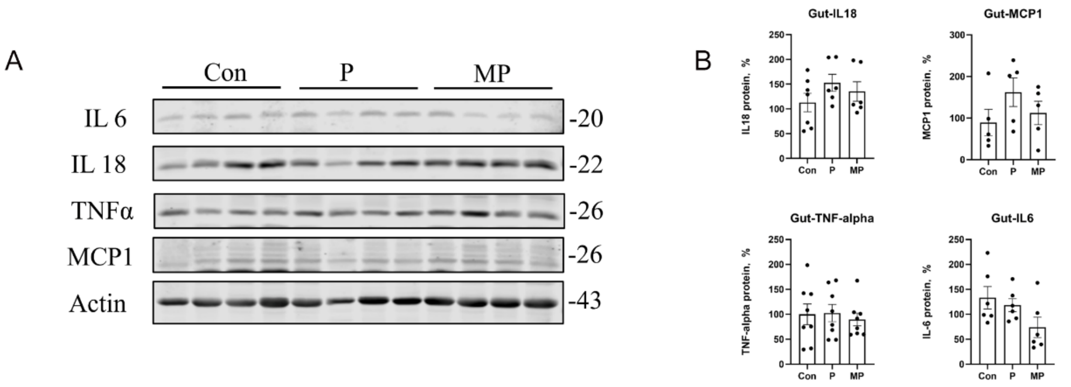

A: Inflammatory protein expression in intestinal tissue; B: Grayscale value.

Figure S3 Mogroside on lung microbiome of broilers with pneumonia induced by $PM_{2.5}$

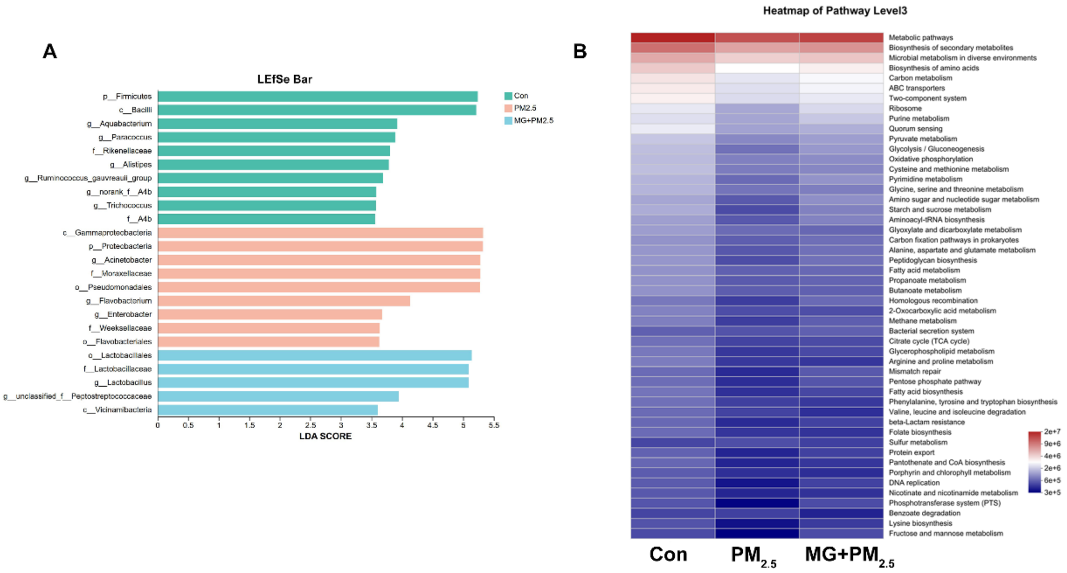

LDA score ≥ 3.5.

Figure S4 BALF Transplantation on Lung Microbiota Composition in $PM_{2.5}$-Induced Dysbiosis

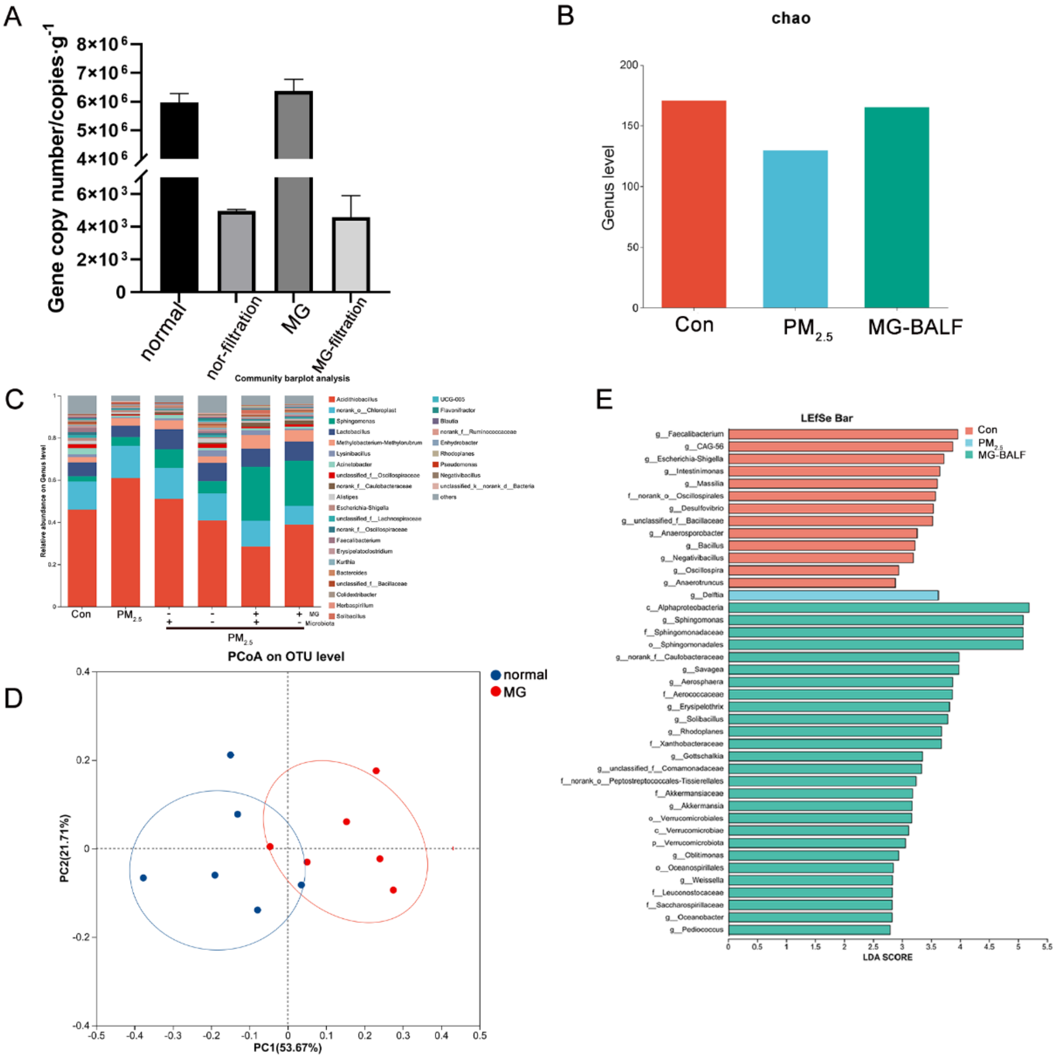

A: Copy numbers of bacterial genes in bronchoalveolar lavage fluid and filtered bacterial lavage fluid. B, α-diversity C: Genus community of all group; D: Pcoa between normal and MG-BALF; E: LDA socre.

Figure S5 BALF Transplantation on Metabolic Alterations in $PM_{2.5}$-Induced Dysbiosis

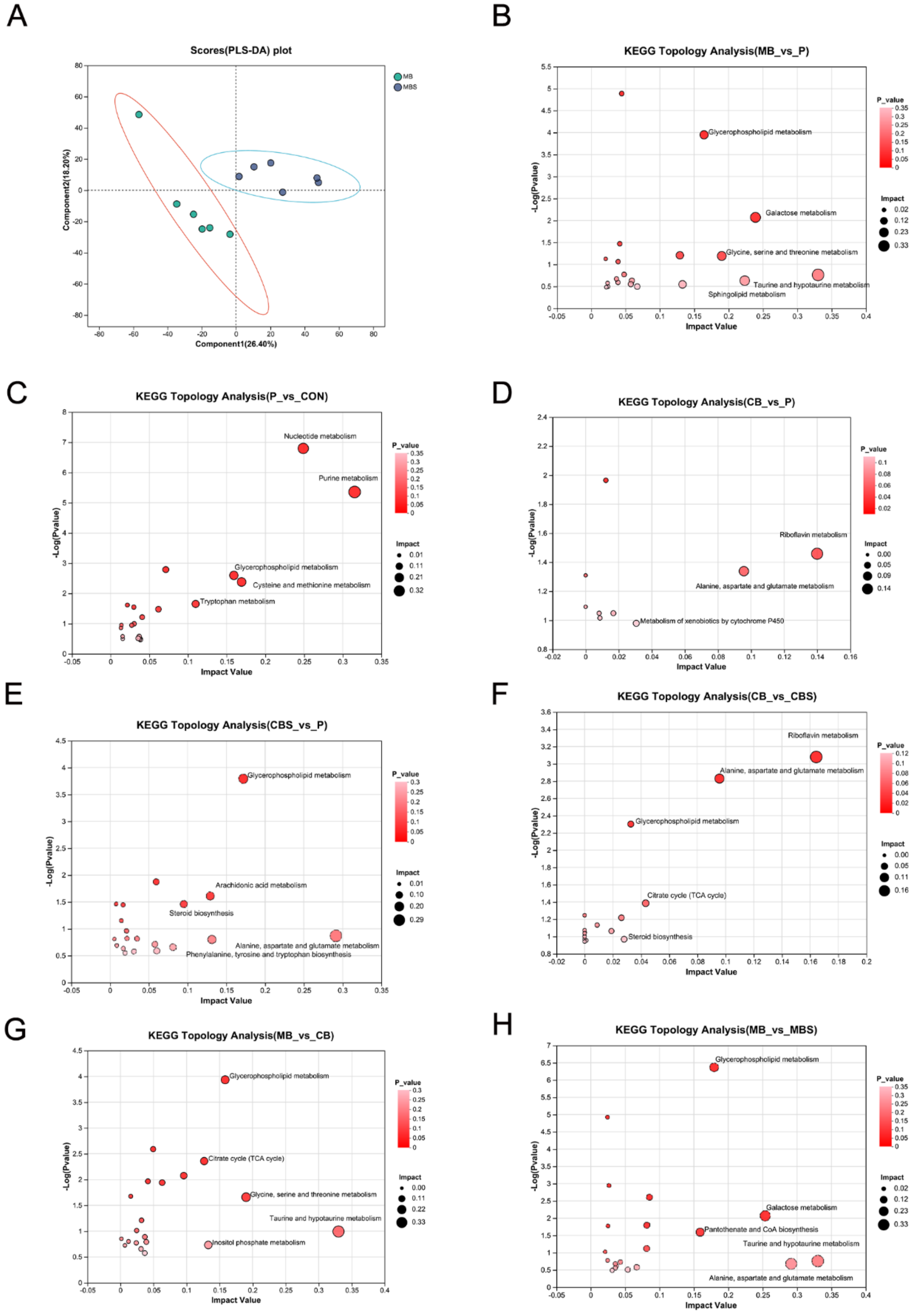

A, B: PLS-DA scores. C-H: KEGG topology analysis.

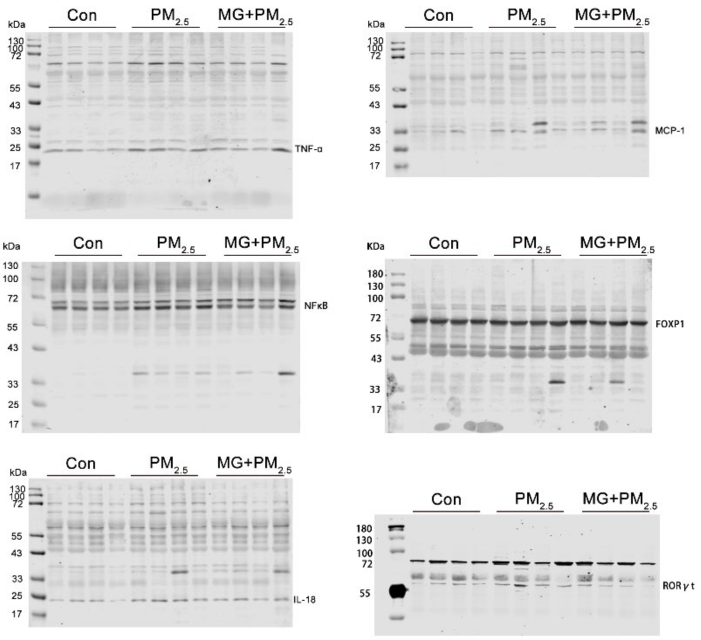

For Trial 1, we performed Western blotting using full, uncut membranes.

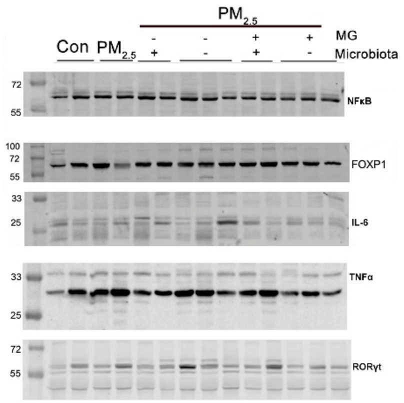

For Subsequent Trials, Having precisely established the band distributions, we adopted a physical membrane-cutting strategy for all subsequent biological replicate experiments. Prior to blocking, the transferred membranes were physically cut into horizontal strips encompassing the specific molecular weights. This protocol optimization was strictly necessitated by the extreme cost and limited commercial availability of high-quality, chicken-specific primary antibodies. Cutting the membrane allows us to drastically cut down on antibody use while simultaneously detecting the target protein and loading control from the exact same gel run, avoiding the signal degradation often caused by harsh stripping protocols.
